# Combinative effects of thinning and prescribed burning on fuel reduction and soil arthropods: A case study in a Mediterranean pine forest

**DOI:** 10.1002/ece3.70141

**Published:** 2024-09-13

**Authors:** Pauline Longeard, Mathieu Santonja, Fréderic Morandini, Marc Gibernau, Sugahendni Nadarajah, Pauline Belliard, Nathalie Feignier, Antonella Massaiu, Marie‐Cécile Andrei‐Ruiz, Lila Ferrat

**Affiliations:** ^1^ UMR CNRS 6134, Sciences Pour l'Environnement University of Corsica Corte France; ^2^ Aix Marseille Univ, Avignon Univ, CNRS, IRD, IMBE Marseille France; ^3^ Office National Des Forêts – Unité DFCI Sainte‐Lucie de Porto‐Vecchio France; ^4^ Office de l'Environnement de la Corse – Observatoire, Conservatoire Des Insectes de Corse Corte France

**Keywords:** fire dynamics, forest management, *Pinus laricio*, slashes, soil fauna

## Abstract

Wildfire pressure involves today to implement silvicultural practices that provide a good compromise between reducing fire risk and maintaining ecological functioning. Thinning reduces tree density and low branches, but results in the deposition of a considerable biomass of woody debris on the ground (up to 4800 g m^2^ in this study). They can be eliminated by prescribed burning, but this raises questions about the fire intensity that can be generated and the impact on soil fauna. We undertook the monitoring of a thinning and prescribed burning operation, separated and combined, in November 2020, in a *Pinus laricio* stand prone to fire risk, located in Bavella, Corsica. Fuel load was determined, and temperature measurements in the soil were performed using K‐type thermocouples. Soil arthropod populations were monitored using pitfall traps, in particular Collembola, Acari, Aranae, and Coleoptera. The combination of thinning and burning resulted in a fire intensity of 75.8 versus 8.4 kW m^−1^ for burning alone. Maximum temperature rise measured at −2 cm below the surface was less than 5°C for both treatments. The combination of thinning and burning did not result in higher fire intensity at ground level than burning alone, and the soil showed high insulation capacity. Most of the woody debris that burned was small‐diameter, and large‐diameter debris remained unconsumed. This burning, performed during a period of low biological activity, had no effect on soil arthropods, and the presence of large debris may have provided refuge areas. Collembola group was the faster to recover, and were followed by cohorts of predators in summer, especially Acari. Our results suggest that a combination of burning and thinning in autumn may be beneficial for fire prevention. However, the decomposition of woody debris in relation to fire risk, and the occurrence of pests after these treatments need to be monitored.

## Graphical abstract

Soil arthropods of *Pinus laricio* forests are little affected by thinning and prescribed burning conducted in autumn. Low‐intensity burns preserve soil‐dwelling organisms, and unconsumed wood serves as a refuge area. The combination of thinning and burning appears to be beneficial in reducing the fire risk and protecting soil arthropods, notably Collembola and Acari.

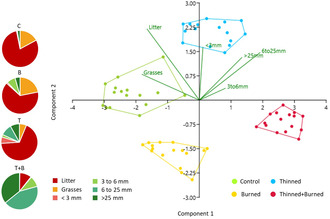

## 1 INTRODUCTION

In recent years, the problem of wildfires has globally expanded and intensified (Duane et al., 2021). Their impacts on biogeochemical cycles, biodiversity, and economic and social activities are well‐documented (Riera et al., 2007). In southern France, forest fire prevention measures have been reinforced by long‐term forest management plans (e.g., PFFENI: fire protection plan for forests and natural areas). These management plans, drafted by an inter‐service working group (including forest department and firefighting units), define the general policy for the protection of forests against wildfires (DRAAF, 2023). They improve public information and awareness of the fire risk, but their main objective is to reduce the fire risk through the installation of equipment, surveillance, and specific legislation. Located in the Mediterranean basin, Corsica is a mountainous island with important slopes, agricultural abandonment, and increasing forest‐habitat interfaces, which make it highly vulnerable to forest fires (Aquilué et al., 2020; Tecimen et al., 2021). The afforestation rate on the island has risen from 15 to 20% in 1980 to over 60% in 2022 (IGN, 2023), making Corsica one of the 10 most forested regions in France, with 577,000 ha of forest. Pine forests are particularly affected by fires, accounting for 19% of the total area burnt on the island over the past 30 years. Pine forests cover 75,408 ha in Corsica, and 18% of these forests were covered by fires between 1989 and 2022. Reducing vegetation density through thinning (Vilà‐Vilardell et al., 2023) or prescribed burning (Stephens & Moghaddas, 2005) reduces horizontal and vertical forest connectivity (Agee & Skinner, 2005) but also aims to eliminate intraspecific competition and makes resources more accessible to the most vigorous trees (Palahí et al., 2003). In this way, the risk and severity of forest fires are greatly reduced for the next fire‐prone seasons (Espinosa et al., 2020; Fernandes & Botelho, 2003).

Thinning operations leave large amounts of woody residue on the ground, which paradoxically increases the fire hazard (Banerjee, 2020; Kalabokidis & Omi, 1998). Prescribed burning is therefore used to remove these woody residue (Schmidt et al., 2008). This technique is mainly used for fire prevention in hard‐to‐reach areas and on steeply sloping terrain, as it saves time and avoids logistical and cost constraints. On the other hand, after decades of fire exclusion across forest ecosystems, the reintroduction of fire allows ecosystem renewal by increasing floristic and habitat diversities (Fernandes et al., 2013; Prichard et al., 2017). However, it remains a controversial approach worldwide (Hunter & Robles, 2020; North et al., 2015), as it is often blamed for causing pollution and carbon dioxide release (Prunicki et al., 2019), reducing biodiversity (Keeley, 2002; Lazarina et al., 2019), and increasing ecosystem disturbance (Ashby & Heinemeyer, 2019; Dey & Schweitzer, 2018; Harrington, 2012; Ryan et al., 2013).

Numerous studies have been conducted to quantify the effects of fire‐related heat stress on trees (synthesis in Ferrat et al., 2021), understory vegetation (Castro Rego et al., 2021), and soil physicochemical properties (Boyer & Miller, 1994; Fernández et al., 2008; Gundale et al., 2005; Vilà‐Vilardell et al., 2023). However, to our knowledge, much less attention has been given to soil arthropods, despite their key implications in organic matter decomposition and nutrient recycling (Hättenschwiler et al., 2005; Santonja et al., 2017). Some studies have focused on the effects of forest thinning (Wikars & Schimmel, 2001) and burning (Apigian et al., 2006) on soil arthropod communities (Grodsky et al., 2018), but very few have focused on the combination of both thinning and burning on soil arthropod communities (de Groot et al., 2016; Eckert et al., 2023). As the combination of mechanical thinning and prescribed burning is increasingly seen as a potentially good management approach to mitigate fire risk and drought stress (Vilà‐Vilardell et al., 2023), it seems essential to further investigate these management methods and the different impacts they may have (Jandl et al., 2019).

In November 2020, the French National Forest Office (ONF) fire‐fighting unit carried out its first mixed silvicultural operations to secure the Col de Bavella in stands of *Pinus nigra* subsp. *laricio* (Poir.) Maire var. *corsicana* (Loudon). This area is a strategic point for fire‐fighting, as its treatment protects the whole of the Bavella massif (Corsica, France), which includes important ecological (presence of mouflon (*Ovis aries musimon*) and sitelle (*Sitta whiteheadi*), endemic species) and tourist issues. As the thinning operations had resulted in the deposition of several thousand g m^−2^ of woody debris on the ground, serious concerns were expressed about the intensity of the subsequent prescribed burning that would follow and its impact on soil organisms in cumulative treatments. In collaboration with operational teams, we set up 4 monitoring plots in the stand to understand the effect of single and cumulated thinning and burning on fire behavior and soil fauna. Our hypothesis was that the heavy accumulation of fuel could lead to a fire of higher intensity with stronger negative impact on soil‐ and litter‐dwelling arthropod populations. This opportunity allowed us to set up a multidisciplinary monitoring study combining the characterization of the burning, the thermal measurements in the soil, and the monitoring of arthropod populations.

## 2 MATERIALS AND METHODS

### 2.1 Site description

**FIGURE 1:**
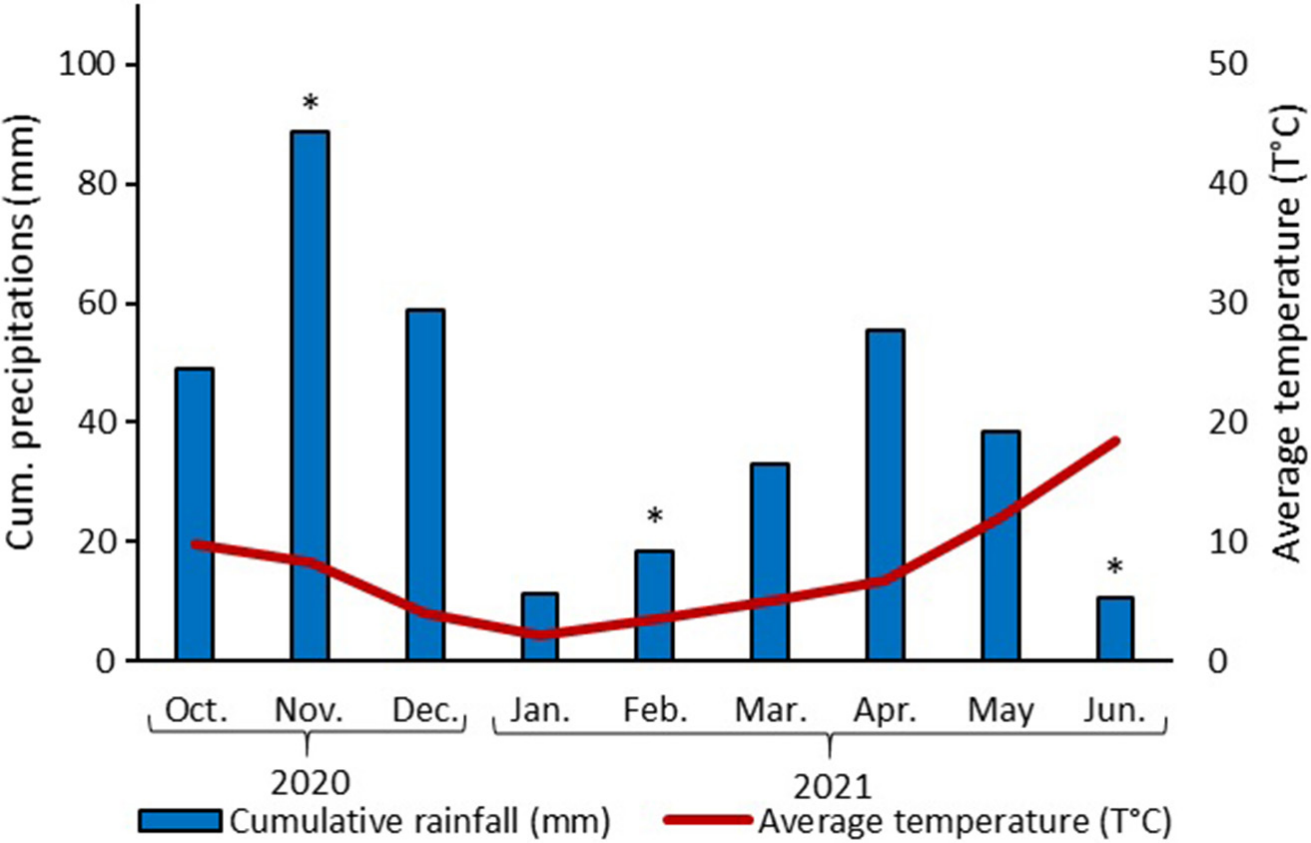
Ombrothermic diagram (with the scale of the precipitation data at twice that of the temperature data; Emberger et al., 1963) of the study site during the period from October 2020 to June 2021 (e.g., Infoclimat). The blue bar stand represents monthly precipitation (mm), the red line represents the mean monthly temperature (°C), and the stars represent the sampling periods.

The study was carried out in a 30‐year‐old *Pinus laricio* forest stand located in Bavella, South Corsica, France (N 41° 47′29.337″, E 9° 13′27.605″) at an altitude of 1218 m (Table 1). This forest is managed by the French National Forest Office (ONF). Trees are 9.1–12.7 m high and 12.4–18.1 cm DBH (Diameter at breast height). The understory is sparse and consists of *Pteridium aquilinum* (L.) Kuhn and *Juniperus communis* (L.), and the herbaceous layer is mostly represented by *Brachypodium pinnatum* (L.) P. Beauv. The soil in this forest is classified as “leptosol” (IUS World Reference Base), consisting of granitic rocks (graniodiorites and alkaline granites) on a siliceous substrate. The slope is approximately 10–20% with a western exposure. The meteorological trends are similar during the study, with hot, dry summers and mild winters (average temperature during the study: 7.8°C; temperature min: −4.5°C; temperature max: 22.5°C, 529.8 mm of total rainfall) (Figure 1).

**TABLE 1:** Stand characteristics of the four studied stands.

Stand	Control	Burnt	Thinned	Thinned+burnt
Treatment	No thinning, No burning	No thinning, burning: 10.11.2020	Thinning: 10.2019 No burning	Thinning: 10.2019 burning: 10.11.2020
Location	41.794177 N; 9.223644 E	41.794517 N; 9.224094 E	41.790685 N; 9.223833 E	41.791053 N; 9.224819 E
Surface (m^2^)	625	625	625	625
Elevation (m)	1218	1218	1218	1218
Mean slope (%)	10	10	20	20
Exposition	W	W	W	W
Basal area (m^2^ ha^−1^)	83.5	82.5	64	63
Mean tree density (tree.ha^−1^)	4200	4272	3333	3024
Mean tree height (m)	10.28	9.14	13.84	12.72
Mean tree DBH (cm)	12.89	12.44	18.4	18.12
Soil type	Granitic	Granitic	Granitic	Granitic

**FIGURE 2:**
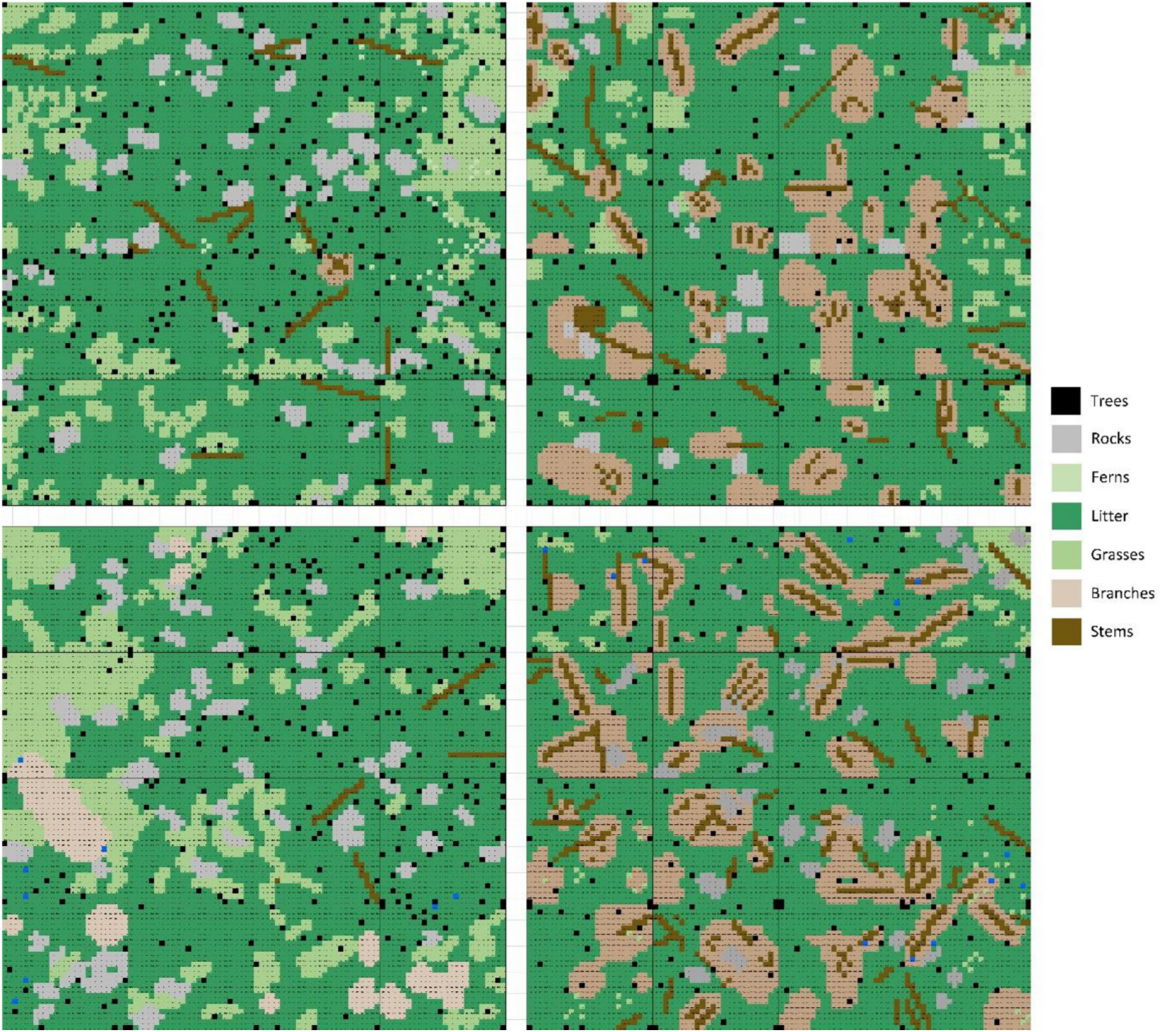
Schematic representation of the fuel load (litter, branches, stems) into the stands before prescribed burning in October 2020.

**FIGURE 3:**
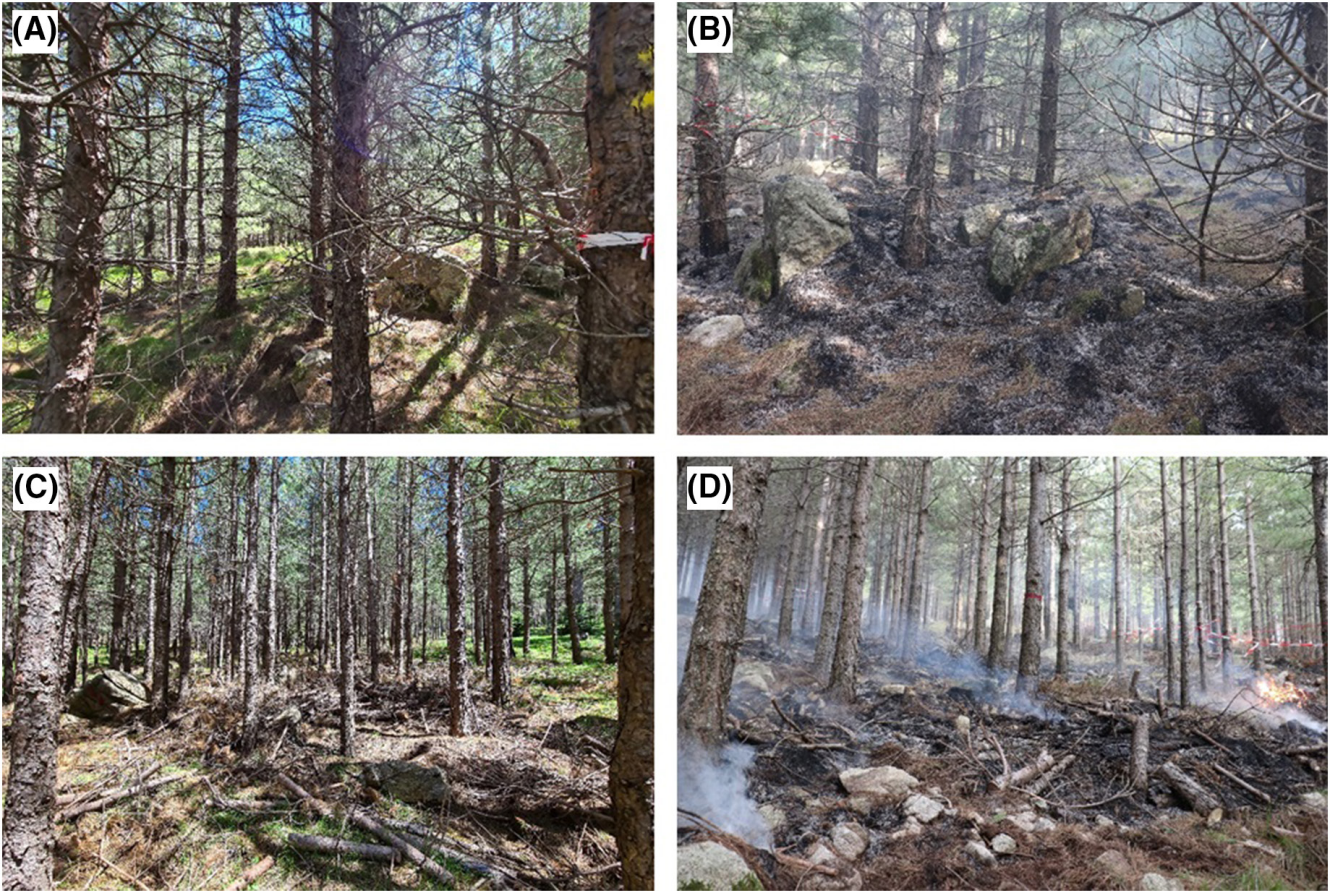
Photos of the untreated and burnt plot before (A) (similar to the control) and after (B) the prescribed burning, and of the treated and burnt plot before (C) (similar to the thinned) and after (D) prescribed burning.

Four plots of 625 m^2^ were established to consider a control, a burning, a thinning, and a thinning+burning treatments (Figure 2). Each plot was therefore assigned to a type of treatment. The four plots were separated by a 5‐meter buffer zone. The control plot received no management treatment, the thinned and thinned+burnt plots were thinned in October 2019. About 20% of the trees were eliminated by mechanical thinning, and the lower branches of the remaining trees were removed. Thinning debris were left as they were, without shredding, resulting in a large number of branches and stem fragments heterogeneously distributed on the ground (Figure 2). Finally, burnt and thinned+burnt plots received prescribed burn treatment in November 2020 (Figure 3). Each plot was subdivided into 16 subplots (39 m^2^ area) to accurately estimate the fuel type and fuel load and to collect arthropods.

### 2.2 Pre‐ and postburning fuel characterization

Litter and herbaceous biomass and cover were quantified on each subplot over an area of 0.25 m^2^ (5 replicates randomly spaced per subplot). Woody fuel load was classified as a function of diameter (<3, 3–6, 6–25 mm, and stems) according to the protocol of Tihay‐Felicelli et al. (2016), which demonstrates the contribution of each class to fire dynamics. The samples were oven‐dried at 60°C for 48 h, and the dry fuel load was recorded. The fuel load was expressed in g m^−2^ of dry weight.

### 2.3 Prescribed burning and instrumentation

**FIGURE 4:**
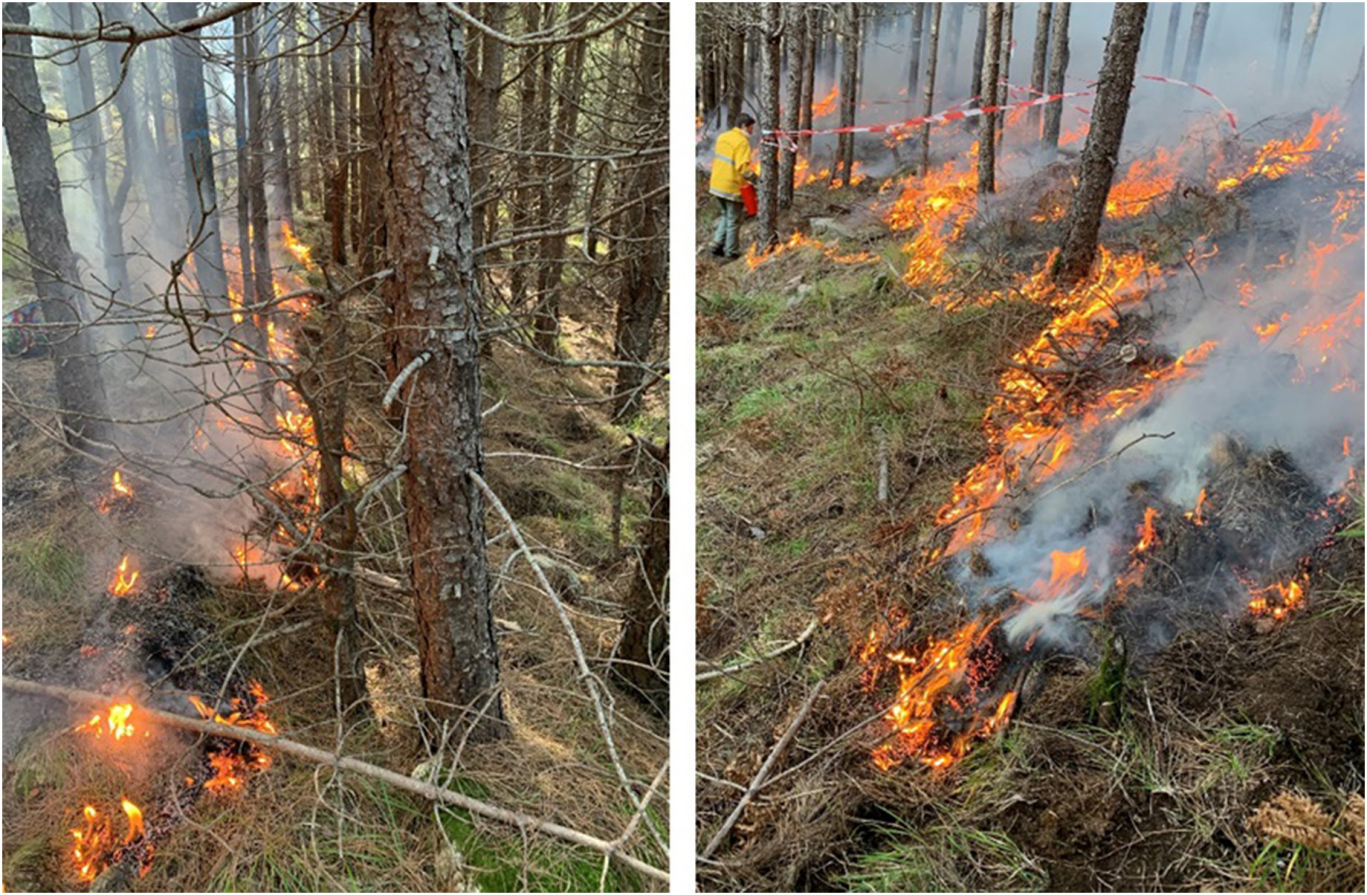
Photographs of the prescribed burning conducted across Burnt and Thinned+Burnt plots.

The prescribed burning was carried out by trained forest managers (ONF, DFCI team) on November 10, 2020, between 11:30 and 14:00 (Figure 4). On the burnt plots, the ignition line was performed at the upper edge of the slope using drip torches. The fire spread was controlled on the edges of the plot using fire rakes. Backpack pumps were also used in some rare cases to prevent the initiation of fire transition toward the crown of the pines.

The fireline intensity during the prescribed burning was estimated according to Byram's criterion (1959). It is defined as the rate of heat release per unit time per unit length of fire front. The fireline intensity is a widely used measure in forest fire applications; it helps to evaluate with simple metrics, the effects of fuel treatment on fire behavior (Fites‐Kaufman & Henson, 2004), to establish limits for prescribed burning (McArthur, 1967), and to assess fire impacts on ecosystems (Hammill & Bradstock, 2006). It is given by:

$$I=r\;w\;H\;\left(1\right)$$

where I (kW m^−1^) is the fireline intensity, r (m s^−1^) is the rate of spread of the fire, w (kg m^−2^) is the weight of the fuel consumed per unit area in the active flame front, and H (kJ kg^−1^) is the heat yield, which can be fixed to a nominal value of 18,000 kJ kg^−1^ (Alexander, 1982). The rate of spread was evaluated from the time taken for the firefront to travel 1 m.

Prior to burning, a series of K‐type thermocouples (chromel‐alumel thermocouples, Omega Engineering, Inc. Stamford, CT, USA) were placed under each fuel categories (litter, branches, logs) over the burnt and thinned+burnt stands. For each one, 4 thermocouples were buried underground at +4, 0, −2, and −4 cm from the ground surface. The extension cords were buried at 20 cm depth, 2 m outside the plots, resulting in a minimal and very local disturbance of the soil in the plots.

The thermocouple body was insulated using multilayer insulation materials based on ceramic to prevent measurement bias due to heat conduction along the stainless‐steel sheath to the junction. Extension cables were buried underground to prevent thermal degradation during the fire. The entire set of thermocouples was plugged into 4 synchronized battery‐powered data loggers (CR3000, Campbell Scientific Ltd., Loughborough, UK) located outside the burn area. Due to the large area of the plots, several data loggers were used to minimize the length of the extension cables. The data were recorded for more than 1 h to observe the heat conduction from the surface through the soil depth after the fire had passed. The sampling rate was 1 Hz.

### 2.4 Arthropod collection

Soil arthropods were sampled after the burning on all 64 subplots in November 2020, March 2021, and June 2021 using 10 cm diameter × 6 cm high pitfall traps installed flush to the soil, which are considered flow traps for arthropods (Perry et al., 2018). Pitfall traps, one‐third filled with water and a few drops of neutral soap, were set for 7 days. Collected arthropods were stored in 70% ethanol, counted, and identified to class or order levels by using a binocular microscope in the laboratory. Diptera were excluded due to their ability to fly as adults, making the influence of fire on these groups highly variable (EL Khayati et al., 2023; Thompson et al., 2022). Dividing each plots into 16 subplots enables us to study the evolution of the Collembola and Acari communities in a more precise, localized way, as their prospected area is relatively restricted due to their small size and limited movement ability (Kuznetsova, 2007).

### 2.5 Data analysis

All statistical analyses were conducted using R software (version 4.2.3).

We compared the fuel load on the different plots using multiple comparisons (*kruskall_test* function in the *rstatix* package) to check that the untreated and treated plots were identical. We then performed the same comparisons after the plots had been burnt to see if the fire had modified the fuel load. We performed PCAs to visualize these differences using the pca function in the FactoMineR package.

We used a generalized linear mixed model (*glmer* function in the *MASS* package) with a quasi‐Poisson distribution, followed by post hoc multiple comparisons ANOVA function in the emmeans package, to evaluate how forest management, season, and their interaction affected total arthropod abundances, as well as Collembola, Acari, Coleoptera, and Spider abundances. These four groups were selected because they represent over 95% of the total sampling. Forest management treatments (Control, Thinning, Burning, and Thinning+Burning) and season (autumn, spring, summer) were used as fixed factors and pitfall trap locations as a random factor in the models.

## 3 RESULTS

### 3.1 Preburn fuel characteristics

On average, herbaceous cover and biomass were greater in the control stand than in the thinned stand (78% less for herbaceous plants, 17% less for litter, Wilcoxon tests, *p* < .05, Table 2).

**TABLE 2:** Fuel load characteristics of pre‐ and postburn stands.

		Control	Burnt		Thinned	Thinned+burnt	
		Preburn	Preburn	Postburn	Preburn	Preburn	Postburn
Litter	Cover (%)	74.6 ± 1.79	68.5 ± 3.51	42 ± 4.3	65.5 ± 1.96	61.7 ± 2.25	28.3 ± 3.69
	Biomass (g m^−2^)	129.1 ± 4.74	211.1 ± 8.27	131.51 ± 12.8	142.9 ± 4.08	130.4 ± 4.38	59.8 ± 7.64
Grasses	Cover (%)	16.1 ± 3.1	19.6 ± 4.08	14.2 ± 3.55	6.1 ± 1.84	1.8 ± 1.32	0.839 ± 0.607
	Biomass (g m^−2^)	31.2 ± 6.00	35.1 ± 7.32	25.5 ± 6.36	11.2 ± 3.38	3.06 ± 2.25	1.42 ± 1.03
<3 mm	Cover (%)	0.488 ± 0.1	2.74 ± 1.13	1.78 ± 0.7	5.42 ± 0.5	6.67 ± 0.4	2.93 ± 0.4
	Biomass (g m^−2^)	5.5 ± 5.1	52.5 ± 21.7	34.2 ± 13	366.6 ± 70.0	448.2 ± 95.9	195.7 ± 24.2
3–6 mm	Cover (%)	0.118 ± 0.1	1.2 ± 0.5	1.2 ± 0.5	2.1 ± 0.2	2.61 ± 0.2	2.61 ± 0.2
	Biomass (g m^−2^)	2.2 ± 2.2	24 ± 9.9	24 ± 9.94	147.4 ± 14.2	180.2 ± 12.6	180.2 ± 12.6
6–25 mm	Cover (%)	0.1 ± 0.1	0	0	9.8 ± 0.9	11.9 ± 0.8	11.9 ± 0.8
	Biomass (g m^−2^)	10.3 ± 10.3	0	0	695.4 ± 67.1	850.3 ± 59.5	850.3 ± 59.5
> 25 mm	Cover (%)	2.2 ± 0.5	0.7 ± 0.5	0.7 ± 0.5	7.8 ± 0.6	9.8 ± 0.7	9.8 ± 0.7
	Biomass (g m^−2^)	1029.6 ± 268	331.4 ± 226	331.4 ± 226	2636.4 ± 288	3361.5 ± 294	3361.5 ± 294

*Note*: Data are mean values ± SEs; *n* = 16.

Conversely, woody residue from stems and branches on the ground dominates the thinned plot with more than 4000 g m^−2^ (Table 2). The distribution of the particle diameter classes is organized as follows: 3–6 mm diameter: 2%; 6–25 mm: 10%; >25 mm: 8% (Table 2). This distribution was similar for between the burnt and unburnt plots (Wilcoxon tests, *p* > .05).

**FIGURE 5:**
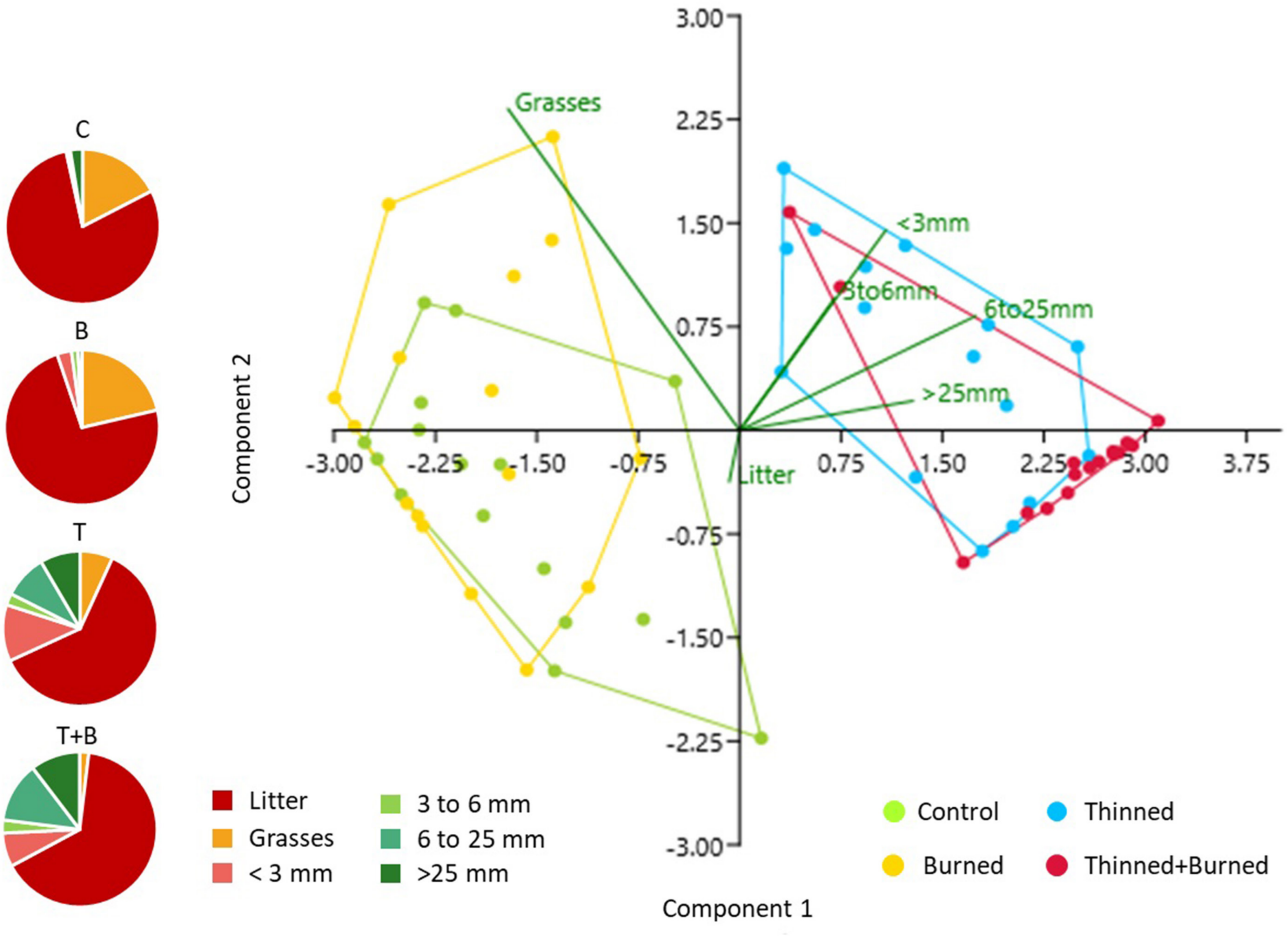
Principal component analysis of fuel load, repartition of the percentage of cover within the different types of particles (grasses, litter, <3, 3–6, 6–25, >25 mm) before burning. C = control, T = thinned, B = burned, and T + B = thinned+burned stands.

**FIGURE 6:**
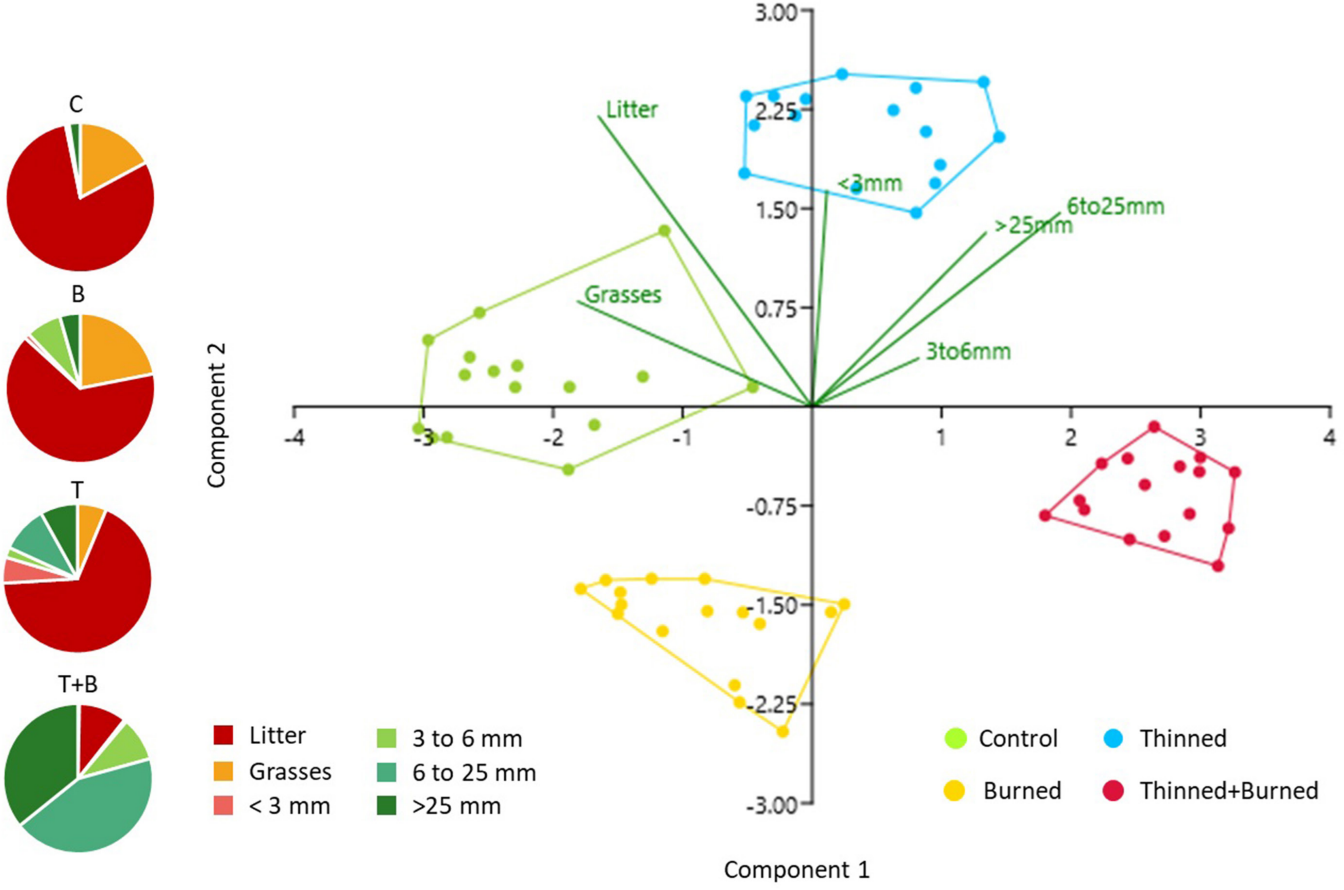
Principal component analysis of fuel load (litter, branches, stem), repartition of the percentage of cover within the different types of particles (grasses, litter, <3, 3–6, 6–25, >25 mm) after burning.

PCAs represent plots according to their fuel load (Figure 5). Before burning, it is possible to differentiate unthinned and thinned plots. After burning, all managed plots were different (Figure 6). Axes 1 and 2 account for approximately 90% of the variance in the fuel load data. Prior to burning, the unthinned plot is mainly characterized by the presence of litter and grasses (*Brachypodium pinnatum*, the other species represent less than 10% of the cover) and differs from the thinned plot, which is characterized by the presence of particles between 3 and 6 mm, resulting from the thinning operation.

### 3.2 Prescribed burning characterization

The prescribed burning was conducted under low wind conditions (<10 km/h). The mean air temperature and relative humidity were 12°C and 60%, respectively. The last rain event occurred 2 days before the experiment. The average moisture content of the OH horizon was 32% and that of the A horizon was 20%.

To minimize the fire intensity and tree damage at the base of the pine stems, the prescribed burning was conducted downslope, following local regulations. The main ignition line was lit at the top of the slope, and the fire spread downslope. Combined with the large relative humidity of air and soil in this season, this intentionally created a low‐intensity backing fire spreading downslope. Secondary ignition points were also created using drip torches when flameout occurred.

**FIGURE 7:**
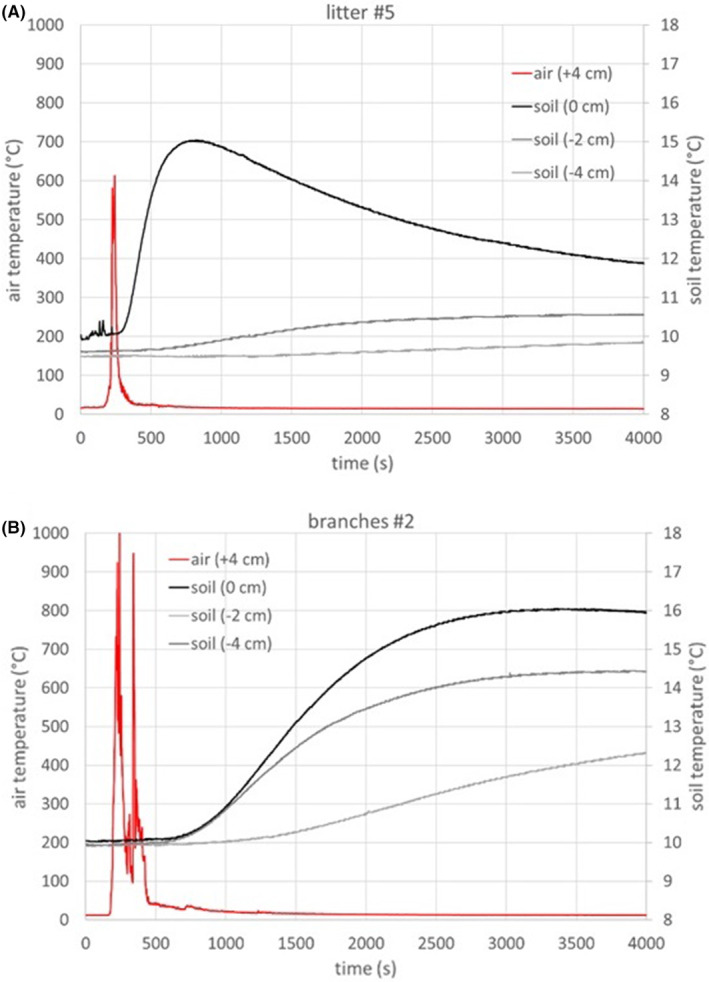
Time evolution (s) of the air and soil temperatures (°C) at different depths recorded during the prescribed burning across (A) litter fuels on Burnt plot and (B) branch fuels on Thinned+Burnt plot.

On both Thinned and Thinned+Burnt plots, for fire spread across pine needles bed, the flames were small and the length was 0.15 ± 0.05 m. On Thinned+Burnt plot, the flame length increased to 0.42 ± 0.20 m when fire spread into cut branch areas (Figure 2). Due to the combined effects of wind and slope, the flames were tilted toward the burnt area (Figure 4). As a result, no transition from the surface to the crown occurred, even for younger pines with bottom branches close to the ground. The fire rate of spread was 0.36 ± 0.18 and 0.54 ± 0.30 m min^−1^ for Burnt and Thinned+Burnt plots, respectively. The fire did not spread across the large piece of cut stems. On both plots, the fire predominantly consumed thin fuel elements like pine needles and herbs. The intensity of the prescribed burning was not high enough to burn the fuel elements present on the ground with diameter greater than 6 mm than remained after the prescribed burn. In particular, the presence of pieces of stem tended to slow down the spread of fire and locally reduced the fire intensity. Conversely, the presence of cut branches with fresh needles on the ground increased the amount of fine fuels prone to ignite and burn. The resulting fireline intensity for Burnt plot was 21.1 ± 13.4 kW.m^−1^. For the Thinned+Burnt plot, it should be noticed that the fireline intensity varied significantly over time and space, influenced by local surface fuel distribution (Figure 2). The fireline intensity was 21.1 ± 13.3 kW.m^−1^ and 72.6 ± 43.8 kW.m^−1^, during fire spread across litter and branches, respectively. A typical example of air and soil temperatures during prescribed burning on is presented in Figure 7 for fire spread across different fuels (litter and branches). The temperatures measured at the different locations vary significantly in terms of both peak levels and duration based on the type of surface fuel present. The maximum temperatures recorded at the different heights and depths below the surface are provided in Table 2 for the different plots. Upon arrival of the fire, the air temperature above the litter increased in the range of 613–1158°C. A significant temperature gradient is observed between the litter surface and the soil. The average values for flame residence times, defined as the duration of exposure to temperatures above 300°C, are also provided in Table 3. Despite the significant temperature increase above the surface during fire spread, there was little conduction of heat deep into the soil. The resulting maximum temperature increase observed over a period exceeding 1 h after the fire spread, measured at −2 cm below the surface was less than 5°C, indicating the good thermal insulation properties of the soil.

**TABLE 3:** Flame residence time and temperature recorded in the burnt stands. Data are mean values ± SEs; *n* = 7.

Stand		Burnt	Thinned + burnt		
Fuel type		Litter	Litter	Branches	Stems
Flame	Residence time (s)	36 ± 11	33 ± 8	57 ± 27	25 ± 1
Litter (+4 cm)	Max temp (°C)	864 ± 162	834 ± 159	911 ± 122	804 ± 9
Humus (0 cm)	Max temp (°C)	146 ± 131	29 ± 19	35 ± 25	63 ± 49
Soil (−2 cm)	Max temp (°C)	11 ± 1	13 ± 1	13 ± 1	11 ± 0
Soil (−4 cm)	Max temp (°C)	11 ± 1	13 ± 1	10 ± 0	10 ± 0

### 3.3 Postburn fuel characteristics

The prescribed burning removed 55% of litter, herbaceous, and particles <3 mm in the Thinned+burnt plot and 33% in the burnt plot (Wilcoxon tests, *p* < .05, Table 2). Particles larger than 3 mm persisted in their entirety after prescribed burning, regardless of the plot considered.

We observed that the litter recovered to the initial level on the burnt plot in less than 8 months (approximately 131.5 g m^−2^ litter on average, Wilcoxon test, *p* > .05).

Before burning, the litter was similar between the burnt and thinned+burnt plots. After burning, the removal of trees on the thinned+burnt plot reduces the amount of needles falling to the soil. The litter reconstitution is therefore greater on the burnt plot.

On the other hand, in the thinned+burnt plot, the litter took longer to recover, averaging 59.8 g m^−2^ in June 2021 vs. 130.4 g.m^−2^ before burning (Wilcoxon test, *p* < .05). The litter is therefore partially reconstituted. By March 2021, the grass cover has returned to its original level, with *Brachypodium pinnatum* still being the dominant species. Ferns also returned, but *Juniperus communis* did not resprout.

### 3.4 Soil fauna characterization

We collected a total of 19,911 arthropods between November 2020 and June 2021 in the 192 pitfall traps installed. Collembola was the most abundant group (13,622 individuals, 68%), followed by Acari (4160 individuals, 21%), Coleoptera (688 individuals, 4%), and Spiders (444 individuals, 2%). The other arthropods represented less than 5% of the collected individuals and included the orders Opiliones, Psocoptera, Lepidoptera, Siphonaptera, Thysanoptera, Hemiptera, Isopoda, Myriapoda, Pseudoscorpiones, Orthoptera, and Dermaptera.

**FIGURE 8:**
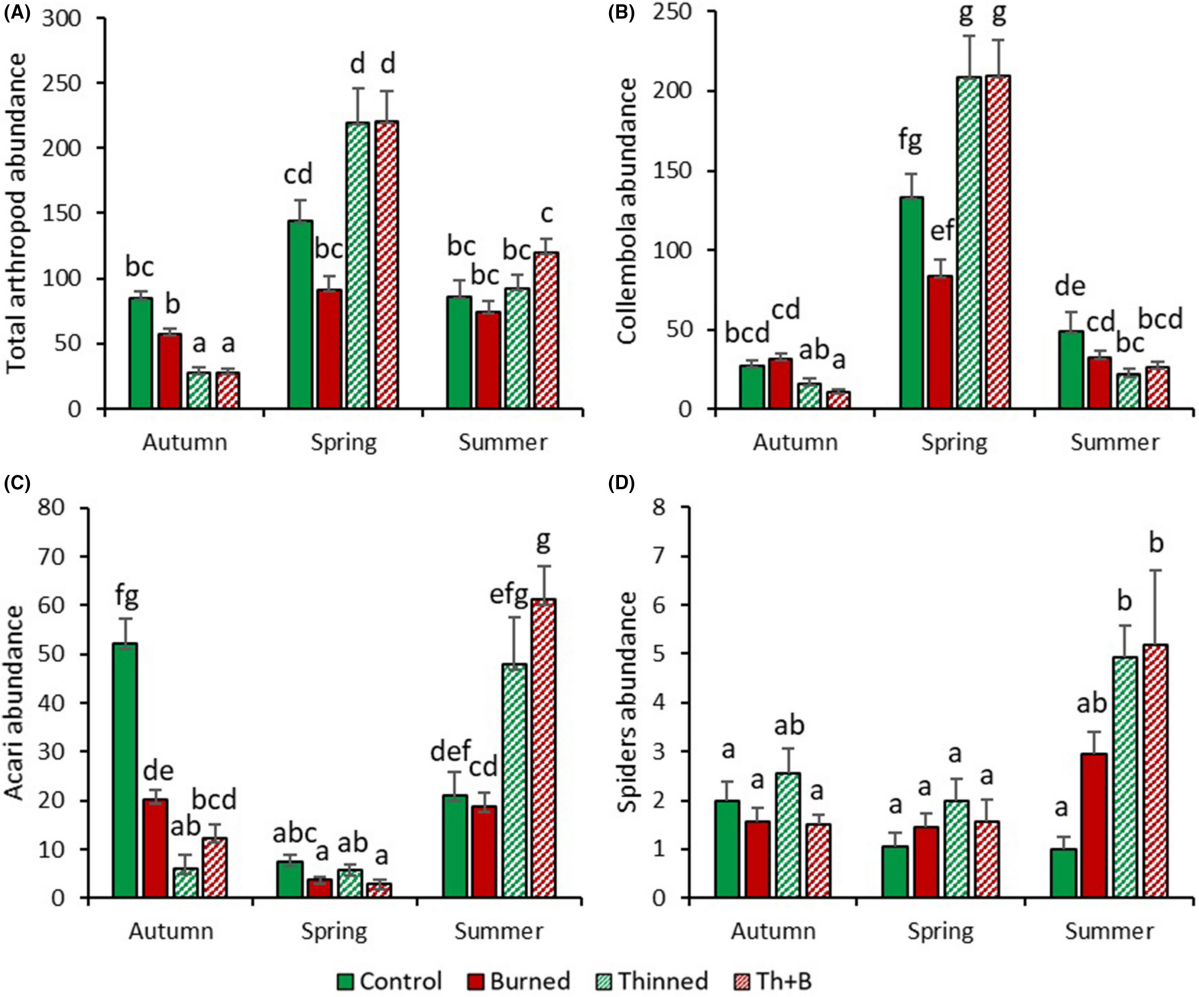
Total arthropod abundance (A), Collembola abundance (B), Acari abundance (C), and spider abundance (D) by season (autumn, spring, summer) according to the season x forest management interaction (Table 4). Arthropod abundance is expressed as number of individuals (nb. ind) collected in the pitfall traps. Values are means ± SE; *n* = 16. Different letters denote significant differences between the different “treatments × seasons” combination.

A strong effect of both forest management and season was observed on the arthropod abundances collected in pitfall traps (Table 4). Except for Coleoptera, forest management and season interacted in their effects on total arthropod abundance and the three other arthropod groups (Table 4). In addition, except for Coleoptera, the effects of the thinned and thinned+burnt treatments on arthropod abundances were similar across taxonomic groups and seasons (Figure 8).

**TABLE 4:** Effects of forest management, season, and their interactions on total arthropod, Collembola, Acari, Coleoptera, and Spider abundances collected in the pitfall traps.

	df	Total		Collembola		Acari		Coleoptera		Spiders	
		*F*	*p*	*F*	*p*	*F*	*p*	*F*	*p*	*F*	*p*
Management	3	6	***	8.5	***	2.4	*	11.4	***	8.2	***
Season	2	65.2	***	245.6	***	28.7	***	117.7	***	17.3	***
M × S	6	7	***	9.46	***	6.43	***	0.4	.82	2.8	*

*Note*: *F* values and associated *p* values (with the respective symbols * for *p* < .01 and *** for *p* < .001) are indicated. Abbreviation: df, degrees of freedom.

For total arthropods, the lowest abundances were observed in the thinned and thinned+burnt plots in autumn 2020 (27 ind/trap), while the highest abundances were observed in the same plots in spring 2021 (220 ind/trap) (Figure 8A). The total arthropod abundance was 36% lower in autumn and summer than in spring in plot control (Figure 8A). No difference between seasons was observed in the burnt plot, while in the thinned and thinned+burnt plots, the arthropod abundance increased according to the gradient autumn<summer<spring (Figure 8A). No difference between forest management treatments was observed in summer, while in autumn, the total arthropod abundance was 68% lower in the thinned and thinned+burnt plots than in the control plot (Figure 8A). Finally, in spring, the total arthropod abundance was 37% lower in burnt plot and 34% higher in thinned and thinned+burnt plots compared to control (Figure 8A).

The Collembola abundance was 83% lower in winter and summer than in spring, regardless of the forest management treatment considered (Figure 8B). No difference was observed between the control and burnt plots across seasons (Figure 8B). Collembola abundance was 52% lower in the thinned and thinned+burnt plots than in the unmanaged plots in autumn and summer (51%), whereas it was 36% higher in these plots in spring (Figure 8B).

Acari abundance was 79% higher in summer and autumn compared to spring in the control and burnt plots, while by contrast, it was higher in autumn compared to spring and summer in the thinned and thinned+burnt plots (Figure 8C). No difference was observed between the control and burnt plots across seasons (Figure 8C). Acari abundance was 82% lower in the thinned and thinned+burnt plots than in the control plot in autumn but only 43% lower in the thinned+burnt plots than in spring. In contrast, Acari abundance was 61% higher in the thinned and thinned+burnt plots than in the control plot in summer (Figure 8C).

No difference in Spider abundance was observed between unmanaged and burnt plots across seasons (Figure 8D). A 62% increase in Spider abundance was observed in summer compared to the two other seasons in the thinned and thinned+burnt plots (Figure 8D). No difference between treatments was observed for Spider abundance in autumn and spring, while Spider abundance was 80% higher in the thinned and thinned+burnt plots than in the control plot in summer (Figure 8D).

**FIGURE 9:**
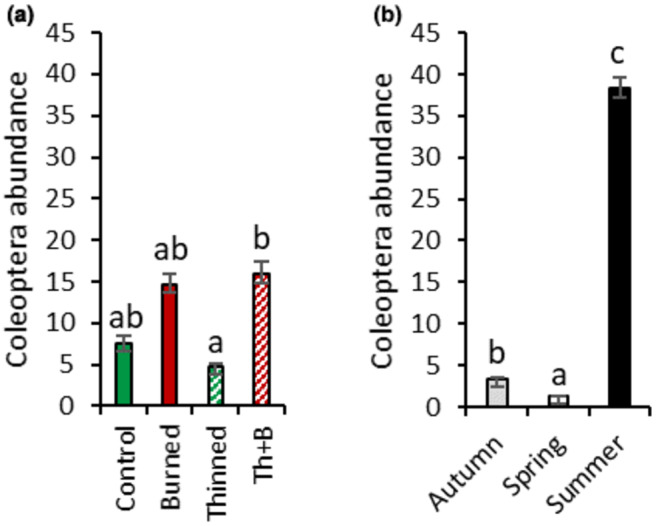
Coleoptera abundance according to season (A) and forest management treatment (B). Coleoptera abundance is expressed as the number of individuals (nb. ind) collected in the pitfall traps. Values are means ± SE; *n* = 64 for (A) and 48 for (B). Different letters denote significant differences between seasons (A) or between treatments (B).

Coleoptera abundance was 94% higher in summer than in autumn and spring (Figure 9A). No difference in Coleoptera abundance was observed between the control and thinned plots, while for the burnt and thinned+burnt plots, Coleoptera abundance increased by 54% (Figure 9B).

## 4 DISCUSSION

### 4.1 Influence of forest management treatments on *Pinus laricio* stands

Thinning allowed the suppression of 20% of the trees, in line with local management plan recommendations. This treatment aimed to reinforce plot growth crown fire protection and maintain protection of the undergrowth (ONF pers. comm., Agee & Skinner, 2005; Johnston et al., 2021), while preserving the landscape aspects that remain important in a touristic area. These guidelines can vary considerably depending on the management objectives and the region considered. For example, Gibson et al. (2020) applied 80% thinning in ponderosa pine forest located in the Valles Caldera National Preserve (New Mexico) to improve tree growth, biological diversity, and fire resistance, whereas Grady and Hart (2006) applied 30% thinning followed by prescribed fire in another ponderosa pine forest located in the Coconino National Forest (northern Arizona) to restore soil processes.

In the present study, thinning resulted in the release of more than 4392 g m^−2^ of woody residue to the ground, leading to concerns about the intensity of the expected burning compared to an unthinned plot. Soler Martin et al. (2017) demonstrated that thinning does not alter the moisture content of fuel or the structure of vegetation cover. In our study, most of the biomass (90%) in the thinned+burnt plots was large‐diameter particles (stems and branches) that did not receive enough heat from the flame to undergo thermal degradation and did not actively contribute to the dynamics of fire spread (Parsons et al., 2018). Litter, herbaceous plants, small branches, and needles (<6 mm) represented a nonnegligible part of the biomass (more than 500 g m^−2^) and contributed to the fire spread. Longer residence times were observed when the flames encountered branch areas (+60% compared to the litter areas in the burnt and thinned+burnt plots), leading to the combustion of all the particles <3 mm. Finally, the fire duration and intensity of this burn were not different from those generally observed for undergrowth burning. Indeed fine fuel loads in the range of 500–1000 g m^−2^ are very common for burning operations under pine forests (Arévalo et al., 2014; Cannac et al., 2009; Rigolot et al., 2012) where only the thinnest diameters of fuel elements (<4 mm) were consumed, even for higher spring burning intensities (Ferrat et al., 2021). It should be noticed that prescribed burning conducted for the present study was in the optimum intensity range of 17–350 kW m^−1^ for which little damage is done to forest trees defined by (McArthur & Cheney, 2015). As a result, 37% of the litter and 27% of the herbs were removed in burnt plot, and 54% of the litter, 95% of the herbs, and 53% of the small branches were removed in plot thinned+burnt.

Despite the slow rate of spread of the main flame front, relatively short residence times of less than 1 min were observed. In addition, the presence of large particles in the woody residue piles hindered the combustion of the finer fuels located underneath, resulting in shorter residence times in stem areas (−30% compared to the litter areas). This fire behavior prevented heat transfer to the soil. As a result, the heating was attenuated in the humus since it did not exceed 146°C. At a depth of 2 cm in the soil, during the burn, no temperature increase was observed. This finding suggests that there was no organic matter combustion or carbon volatilization, which occurred at 300°C and 200°C, respectively (Santín & Doer, 2016).

Studies have already demonstrated the strong insulating properties of soil (see synthesis in DeBano, 2000), regardless of its type. For example, Fernández et al. (2008), and Fajardo‐Cantos et al. (2023), respectively, show that alumi‐umbric regosol and eutric‐cambisol soils can attenuate about 600°C at –2 cm depth during prescribed burns with the same characteristics as ours. This may be due to the thermal protection provided by the presence of a protective layer of litter, combined with poor heat conduction through the mineral soil.

### 4.2 Arthropod responses to season and forest management treatments

#### 4.2.1 Influence of season

In November 2020, arthropod abundance was relatively low in all the plots studied. This observation is related to the low temperatures encountered in autumn (between 9°C and 12°C, Figure 1), which tend to negatively affect soil arthropod activity, which are ectothermic organisms (Gillooly et al., 2001; Vucic‐Pestic et al., 2010). In March 2021, the total number of arthropods increased regardless of the treatment considered. These results are similar to those of other studies that have found a strong seasonal effect on soil arthropod abundance, with higher abundance in spring compared to autumn (Antunes et al., 2009; Auclerc et al., 2019). For example, Antunes et al. (2009) captured two times more individuals in spring compared to autumn (538 vs. 226). In our study, the increase in soil arthropod abundance in spring was strongly related to the increase in Collembola abundance, with milder temperature conditions favoring the Collembola population at this time of year.

The abundances of Coleoptera and Acari increased in June 2021, in contrast to Collembola. First, this may be partly related to increasing temperatures (12°C–19°C), favoring arthropod mobility and plot recolonization. It has been shown that Coleoptera tend to emerge from spring onwards, after a period of winter diapause or hibernation (Andersen, 2011; Borowiec, 2022), and are more affected by an increase in temperature than in humidity (Wardhaugh et al., 2018). Second, an increase in prey availability, such as Collembola, can also explain this increase in predator abundances in June. As Carabidae beetles and Acari Mesostigmata are predators of Collembola (Aupic‐Samain et al., 2021; Bilde et al., 2000), we can hypothesize that their later appearance is mainly due to the availability of their prey. In fact, the increase in these two predator groups was concomitant with a decrease in Collembola abundance.

Finally, the spider abundance remained unchanged between March and June 2021. Spiders are known to be strongly influenced by both abiotic conditions, such as temperature and humidity (Campuzano et al., 2020; Pekár et al., 2015), and prey availability (Riechert & Lockley, 1984). However, the small abundance of collected individuals in the present study limits us from proposing explanations of the observed pattern of Spiders response to seasons.

#### 4.2.2 Impact of thinning

In November 2020, there were 68% fewer arthropods in the thinned than in the control plot, mainly due to 42% fewer Collembola and 88% fewer Acari. Thinning requires a large amount of machinery and the movement of a significant number of managers on the plot while for burning, only three people were present on the plot. The reduction in arthropod abundance observed in November could therefore be related to the immediate disturbance caused by the application of the forest treatment (Edlund et al., 2013). In addition, the arrival of large amounts of nondegraded fuel on the plot may have caused ecosystem instability and slow colonization of these new areas (Eckert et al., 2023).

In March 2021, the total arthropod abundance in the thinned plot was higher than that in the control. This trend is mainly because the thinned plot had 35% more Collembola. The increase in Collembola abundance may be related to habitat restoration and to the presence of dead wood on the site. Numerous studies have shown that dead wood can be used to create diverse microhabitats for saproxylic and nonsaproxylic Collembola (Eckert et al., 2023; Raymond‐Léonard et al., 2020). These habitats could protect these organisms from predators, temperature fluctuations, and drought and provide a readily available food source (Gibson et al., 2022).

Acari and Coleoptera abundances were not influenced by the presence of woody residue at the site. De Groot et al. (2016) found no effect of 50% thinning or clear‐cutting on Coleoptera after 1 year. Jonsell (2008) reported that the high content of secondary metabolites in dead wood could have a negative effect on Coleoptera communities. As the degradation of secondary metabolites can take several years, and as the clearing is relatively recent, it is possible that they may influence plot recolonization by Coleoptera.

In June 2021, the total arthropod abundance in the control and thinned plots was similar, but the community composition was different, suggesting that thinning alters the arthropod community composition. Acari tended to dominate the arthropod population in thinned plot (75% of total abundance). The population of Acari increased in relation to the presence of Collembola, which are among their favorite prey (Aupic‐Samain et al., 2021). In addition, Gwiazdowicz et al. (2011) showed that Mesostigmata Acari was more abundant in highly decomposed wood than in slightly decomposed wood. Oribatid Acari feed on fungal hyphae and dead plant material and to some extent on lichens, mosses, and algae (Erdmann et al., 2012). They may therefore have been preferentially attracted to plots with more dead wood.

The creation of new habitats conducive to web development and predation seems to have been beneficial for Spiders, whose abundance was higher on the thinned plot, regardless of the sampling time considered. However, given the very low numbers of spiders present, it seems important to remain cautious about data interpretation.

#### 4.2.3 Impact of prescribed burning

In the present study, the majority of arthropods were not affected by the burning treatment, regardless of the sampling time considered. Organisms attached to the soil surface and litter (Huebner et al., 2012) may be highly vulnerable to fire (Gongalsky & Persson, 2013; Moretti et al., 2004; Zaitsev et al., 2014), which would explain the lower abundance of Spiders in November 2020, which are very active on the soil surface (Buddle et al., 2000). Conversely, organisms may have moved to protect themselves from the heat; Collembola and Oribatid Acari into deeper soil layers (Gongalsky & Persson, 2013; Paquin & Coderre, 1997), and the most mobile, like Coleoptera, toward the outside of the plots (Malmström, 2012; Moretti & Legg, 2009). In addition, some arthropods, including Coleoptera and Acari, have thick and sclerified cuticles, which can protect them from the high temperatures generated by fire and from subsequent drought conditions (Wikars et al., 2005). These parameters may have contributed to the rapid reappearance of Acari, Coleoptera, and the dominance of Collembola in spring. Only Spiders were more abundant in the burnt plot. Cadena‐Zamudio et al. (2022) showed that the influence and severity of burning, as well as the time after burning, can strongly influence arthropod abundance and recovery (Malmström, 2010).

In our study, the presence of preserved litter (11%) and grasses (4%) and their rapid recovery may have favored the recolonization and dispersion of arthropods into burnt areas, depending on their mobility (Gongalsky & Persson, 2013). The presence of woody residue, the thermal insulation provided by the soil around the roots during the fire, and the regenerative capacity of the aerial parts during rainy periods allow a rapid recolonization of the grasses (Moravec, 1990): in March 2021, 4 months after the burning treatment, the herbaceous plants returned to their preburn cover values on all the plots. This observation is in accordance with Arévalo et al. (2014) and Whisenant et al. (1984), who observed a recovery between 2 and 6 months after fire in meadows and Canary pines (*Pinus canariensis*) forests located in Artenara, Canary Islands.

The small size of the plots (25 m x 25 m) may also have favored the recolonization from peripheral unburnt zones (Larrivée et al., 2008), especially for highly mobile organisms such as Coleoptera (Baars, 1979; de Groot et al., 2016) and Spiders (Thévenard et al., 2004). This very early postfire dominance of Collembola can be explained by the short life cycle of these organisms and their ability to carry out parthenogenesis, which allows them to reproduce rapidly. Moreover, these epigeomorphic and homeomorphic groups particularly benefit from the resources provided by the presence of litter and grass roots and associated mycorrhizal fungi (Gibson et al., 2022). Like Antunes et al. (2009), who studied edaphic macroarthropod communities after a forest fire, we can assume that the impact of prescribed burning on arthropods remains limited, but longer‐term monitoring is needed to confirm these conclusions, as some studies show that the effects on arthropods may be delayed (Çakır et al., 2023), 1 year after the prescribed burning.

#### 4.2.4 Impact of thinning combined with prescribed burning

Contrary to our expectations, the combination of thinning and burning (thinned+burnt) did not have a greater effect on arthropod communities than burning alone. As the piles of stems and branches were not consumed, the fire dynamics were quite the same in the burnt and thinned+burnt treatments. The presence of piles and the preservation of litter underneath them may have increased the number of microhabitats, acting as refuge areas (Cobb et al., 2007) and starting points for area recolonization (Mott et al., 2021; Nadel et al., 2007). We also found that needle litter recovered less quickly in the thinned than in the unthinned plots (131.1 vs. 59.8 g m^−2^). This is obviously due to the reduced canopy cover, but it also reflects the fact that the tree vitality was not affected by burning. In fact, no significant needle scorching was observed after the burns. Litter is an essential element for the development of certain organisms, such as decomposers, which live in the superficial parts of the soil. Litter layer reconstitution, although slower on thinned plots, allows the recolonization of the habitat quite quickly, as needle fall from lower branches tends to increase after a burn (Cannac et al., 2009).

Prescribed burning in autumn rather than spring may have reduced the impact of fire on arthropods since milder temperatures would have reduced the intensity of the burn. Arthropods are also fewer at this time of year, which may have protected the communities from a higher intensity burn, which could have had a greater impact. As shown by Collett (2003) in Australia, spring burning is more intense and reduces organism abundance, whereas autumn burning only alters arthropod activity.

### 4.3 Evaluation of the effectiveness of treatments in reducing fire risk

While the scientific literature on the effects of thinning (Bernes et al., 2015) or burning (Alcañiz et al., 2018) on forests is extensive, very few studies have linked the thermal characteristics of prescribed burns and soil temperature transfer in Mediterranean pine forests, and even fewer have linked the two methods.

Thinning reduces pine density and limits vegetation continuity within the forest, thereby reducing fire risk (Agee & Skinner, 2005). On the other hand, thinning without rapid slash reduction leads to a significant increase in the amount of dead fuel on the ground, which could temporarily increase fire risk. According to the literature, thinning alone is less effective than prescribed burning in reducing wildfire risk but more effective than no treatment (Kalabokidis & Omi, 1998; Stephens & Moghaddas, 2005). Piqué & Domènech, 2018 also demonstrated that the way in which slash is distributed and dispersed on the surface can greatly influence fire behavior. Vilà‐Vilardell et al. (2023) showed that the thinned+burnt plot could make pine forests more resistant to drought and fire by reducing the fuel loads (Agee & Skinner, 2005; Schmidt et al., 2011; Stephens & Moghaddas, 2005), increasing the distance between the canopy and the ground, and eliminating competition for available water in the soil. The constraints of the forest environment, linked to the presence of the canopy and the objectives of maintaining the tree stratum, force operators to control fire at low intensity, whether or not slash is present on the ground.

Our study pointed out very little effect of the management treatments on arthropod communities and even suggested a protective effect of woody residue, which, contrary to our hypotheses, did not increase the fire intensity. Thinning resulted in the release of an important amount of biomass to the ground, most of which was in the form of large‐diameter particles. The substantial presence of small branches and needles that dry out very quickly increases the risk of fire. Burning eliminates these particles smaller than 3 mm, which is particularly interesting as they contribute to the dynamics of the fire. The large woody residues were not consumed by the fire and constitute a very important biomass. They are interesting in the sense that they can form microhabitats and preserve soil moisture, but their degradation (drying out/decomposition) over the years needs to be monitored to ensure that they do not contribute to weakening the plots in terms of fire risk. We also found that needle litter recovered less quickly in the thinned plots. This may be due to the reduced canopy, but it also reflects the fact that the trees were not affected by burning. In fact, no significant needle scorching was observed following the burns.

It is well established in the literature that improperly conducted silvicultural treatments can be counterproductive: indeed, litter reconstitution after a high‐intensity burn, can be very important, just as high‐intensity thinning can lead to understory development over time, rendering these treatments ineffective and having a major impact on the ecosystem. The reconstitution of the litter layer in our study plots was sufficient to not inhibit recolonization by soil arthropods and to not increase the risk of fire in the following summer. The understory did not develop, but the herbaceous layer was reconstituted.

## 5 CONCLUSION

Using the configuration of operations carried out on the Bavella pass, we studied the short‐term effects of fire prevention treatment techniques on soil arthropod communities.

In addition to a marked seasonal effect, we found that thinning can benefit the abundance of some soil organisms (Collembola and Acari).

We measured that burning in autumn can be easily maintained at a low intensity at ground level, even in the presence of a considerable amount of woody debris from previous thinning. Soil organisms appear to be little affected, both by the protection provided by the woody debris and by their low level of activity at the time of burning. Our results also suggest that it may be beneficial to combine burning with autumn thinning or fire prevention, by removing small‐diameter woody debris and litter. However, it seems important to monitor the decomposition of the remaining large woody debris over time, with regard to their potential contribution to fire risk and pest outbreaks.

The results presented here demonstrate the value of transdisciplinary studies to improve forest management strategies. Fire characteristics need to be assessed with complementary metrics to overcome the difficulties due to the strong local variation of the combustible. Monitoring organisms at different trophic levels and with different life cycle lengths helps to provide a reliable indication of soil disturbance from silvicultural treatments.

These results need to be confirmed by monitoring new *P. laricio* study sites, in collaboration with forest managers, to improve our knowledge of these ecosystems and their resilience, for sustainable fire prevention operations.

## CONSENT FOR PUBLICATION

All authors gave their informed consent to this publication and its content.

## AUTHOR CONTRIBUTIONS

**Pauline Longeard:** Conceptualization (lead); data curation (lead); formal analysis (lead); investigation (lead); methodology (lead); writing – original draft (lead); writing – review and editing (lead). **Mathieu Santonja:** Formal analysis (supporting); validation (supporting); writing – original draft (supporting); writing – review and editing (supporting). **Fréderic Morandini:** Conceptualization (lead); formal analysis (lead); methodology (lead); software (lead); writing – review and editing (lead). **Marc Gibernau:** Formal analysis (supporting); writing – review and editing (supporting). **Sugahendni Nadarajah:** Investigation (supporting); methodology (supporting). **Pauline Belliard:** Investigation (supporting). **Nathalie Feignier:** Investigation (supporting). **Antonella Massaiu:** Conceptualization (supporting); methodology (supporting); writing – review and editing (supporting). **Marie‐Cécile Andrei‐Ruiz:** Conceptualization (equal); supervision (equal); writing – review and editing (equal). **Lila Ferrat:** Conceptualization (equal); data curation (equal); formal analysis (equal); funding acquisition (equal); investigation (equal); methodology (equal); project administration (equal); supervision (equal); writing – review and editing (equal).

## FUNDING INFORMATION

This work was funded by the Corsican Region and the French state in the framework of the collaborative project GOLIAT (CPER: 40031).

## CONFLICT OF INTEREST STATEMENT

The authors declare that they have no conflict of interest.

## Data Availability

The datasets generated during and/or analyzed during the current study are available on the following link: https://zenodo.org/records/10908460?token=eyJhbGciOiJIUzUxMiIsImlhdCI6MTcxMjEzNzIyMSwiZXhwIjoxNzI1MDYyMzk5fQ.eyJpZCI6IjhjYmQ0ZGUzLWE1MmUtNDFiYS04M2ZiLWI4YzhiMGYyNTkwZiIsImRhdGEiOnt9LCJyYW5kb20iOiJkMTZlNTZlODMyOTE3NTNiYzE4MWVhN2RhNmI1YTg5MSJ9.mD268KyED_qOyHtsrRtpcbEgXDRhE039Naqfp1zxk3L157mvf5DNva_Vu4fgmTIEnaBkbwugMVqDrFadv8ggsw

## References

[ece370141-bib-0020] Çakır, M. , Akburak, S. , Makineci, E. , & Bolat, F. (2023). Recovery of soil biological quality (QBS‐ar) and soil microarthropod abundance following a prescribed fire in the Quercus frainetto forest. Applied Soil Ecology, 184, 104768. 10.1016/j.apsoil.2022.104768

[ece370141-bib-0001] Agee, J. K. , & Skinner, C. N. (2005). Basic principles of forest fuel reduction treatments. Forest Ecology and Management, 211, 83–96. 10.1016/j.foreco.2005.01.034

[ece370141-bib-0002] Alcañiz, M. , Outeiro, L. , Francos, M. , & Úbeda, X. (2018). Effects of prescribed fires on soil properties: A review. Science of the Total Environment, 613–614, 944–957. 10.1016/j.scitotenv.2017.09.144 28946382

[ece370141-bib-0003] Alexander, M. E. (1982). Calculating and interpreting forest fire intensities. Canadian Journal of Botany, 60, 349–357. 10.1139/b82-048

[ece370141-bib-0004] Andersen, J. (2011). Hibernation sites of riparian ground beetles (Coleoptera, Carabidae) in Central and Northern Norway. Norwegian Journal of Entomology, 15, 799–810.

[ece370141-bib-0005] Antunes, S. C. , Curado, N. , Castro, B. B. , & Gonçalves, F. (2009). Short‐term recovery of soil functional parameters and edaphic macro‐arthropod community after a forest fire. Journal of Soils and Sediments, 9, 267–278. 10.1007/s11368-009-0076-y

[ece370141-bib-0006] Apigian, K. O. , Dahlsten, D. L. , & Stephens, S. L. (2006). Fire and fire surrogate treatment effects on leaf litter arthropods in a western Sierra Nevada mixed‐conifer forest. Forest Ecology and Management, 221, 110–122. 10.1016/j.foreco.2005.09.009

[ece370141-bib-0007] Aquilué, N. , Fortin, M.‐J. , Messier, C. , & Brotons, L. (2020). The potential of agricultural conversion to shape Forest fire regimes in Mediterranean landscapes. Ecosystems, 23, 34–51. 10.1007/s10021-019-00385-7

[ece370141-bib-0008] Arévalo, J. R. , Fernández‐Lugo, S. , García‐Domínguez, C. , Naranjo‐Cigala, A. , Grillo, F. , & Calvo, L. (2014). Prescribed burning and clear‐cutting effects on understory vegetation in a Pinus canariensis stand (gran Canaria). The Scientific World Journal, 2014, e215418. 10.1155/2014/215418 PMC4134779 25147839

[ece370141-bib-0009] Ashby, M. , & Heinemeyer, A. (2019). Prescribed burning impacts on ecosystem services in the British uplands: A methodological critique of the EMBER project. Journal of Applied Ecology, 57, 57–2120. 10.1111/1365-2664.13476

[ece370141-bib-0010] Auclerc, A. , Le Moine, J. M. , Hatton, P.‐J. , Bird, J. A. , & Nadelhoffer, K. J. (2019). Decadal post‐fire succession of soil invertebrate communities is dependent on the soil surface properties in a northern temperate forest. Science of the Total Environment, 647, 1058–1068. 10.1016/j.scitotenv.2018.08.041 30180314

[ece370141-bib-0011] Aupic‐Samain, A. , Santonja, M. , Chomel, M. , Pereira, S. , Quer, E. , Lecareux, C. , Limousin, J. M. , Ourcival, J. M. , Simioni, G. , Gauquelin, T. , Fernandez, C. , & Baldy, V. (2021). Soil biota response to experimental rainfall reduction depends on the dominant tree species in mature northern Mediterranean forests. Soil Biology and Biochemistry, 154, 108122. 10.1016/j.soilbio.2020.108122

[ece370141-bib-0012] Baars, M. A. (1979). Catches in pitfall traps in relation to mean densities of carabid beetles. Oecologia, 41, 25–46. 10.1007/BF00344835 28310358

[ece370141-bib-0013] Banerjee, T. (2020). Impacts of Forest thinning on wildland fire behavior. Forests, 11, 918. 10.3390/f11090918

[ece370141-bib-0015] Bernes, C. , Jonsson, B. G. , Junninen, K. , Lõhmus, A. , Macdonald, E. , Müller, J. , et al. (2015). What is the impact of active management on biodiversity in boreal and temperate forests set aside for conservation or restoration? A systematic map. Environmental Evidence, 4, 25. 10.1186/s13750-015-0050-7

[ece370141-bib-0104] Bilde, T. , Axelsen, J. A. , & Toft, S. (2000). The value of Collembola from agricultural soils as food for a generalist predator. Journal of Applied Ecology, 37, 672–683. 10.1046/j.1365-2664.2000.00527.x

[ece370141-bib-0016] Borowiec, B. G. (2022). Mitochondria on demand for overwintering beetles. Journal of Experimental Biology, 225, JEB243523. 10.1242/jeb.243523

[ece370141-bib-0017] Boyer, W. D. , & Miller, J. H. (1994). Effect of burning and brush treatments on nutrient and soil physical properties in young longleaf pine stands. Forest Ecology and Management, 70, 311–318. 10.1016/0378-1127(94)90096-5

[ece370141-bib-0018] Buddle, C. M. , Spence, J. R. , & Langor, D. W. (2000). Succession of boreal forest spider assemblages following wildfire and harvesting. Ecography, 23, 424–436. 10.1111/j.1600-0587.2000.tb00299.x

[ece370141-bib-0019] Cadena‐Zamudio, D. , Ruiz‐Guerra, B. , Castillo, M. L. , Flores‐Garnica, J. G. , & Guevara, R. (2022). Prevalence of stochastic processes in the fire‐mediated reassemblage of the soil arthropod community of a pine forest. Acta Oecologica, 115, 103834. 10.1016/j.actao.2022.103834

[ece370141-bib-0021] Campuzano, E. F. , Ibarra‐Núñez, G. , Machkour‐M'Rabet, S. , Morón‐Ríos, A. , & Jiménez, M. L. (2020). Diversity and seasonal variation of ground and understory spiders from a tropical mountain cloud forest. Insect. Science, 27, 826–844. 10.1111/1744-7917.12693 31112329

[ece370141-bib-0022] Cannac, M. , Barboni, T. , Ferrat, L. , Bighelli, A. , Castola, V. , Costa, J. , Trecul, D. , Morandini, F. , & Pasqualini, V. (2009). Oleoresin flow and chemical composition of Corsican pine (*Pinus nigra* subsp. *laricio*) in response to prescribed burnings. Forest Ecology and Management, 257, 1247–1254. 10.1016/j.foreco.2008.11.017

[ece370141-bib-0023] Castro Rego, F. , Morgan, P. , Fernandes, P. , & Hoffman, C. (2021). Fire effects on plants, soils, and animals. In F. C. Rego , P. Morgan , P. Fernandes , & C. Hoffman (Eds.), Fire science: From chemistry to landscape management (pp. 259–318). Springer International Publishing. 10.1007/978-3-030-69815-7_9

[ece370141-bib-0024] Cobb, T. P. , Langor, D. W. , & Spence, J. R. (2007). Biodiversity and multiple disturbances: Boreal forest ground beetle (Coleoptera: Carabidae) responses to wildfire, harvesting, and herbicide. Canadian Journal of Forest Research, 37, 1310–1323. 10.1139/X06-310

[ece370141-bib-0025] Collett, N. (2003). Short and long‐term effects of prescribed fires in autumn and spring on surface‐active arthropods in dry sclerophyll eucalypt forests of Victoria. Forest Ecology and Management, 182, 117–138. 10.1016/S0378-1127(03)00009-4

[ece370141-bib-0027] de Groot, M. , Eler, K. , Flajšman, K. , Grebenc, T. , Marinšek, A. , & Kutnar, L. (2016). Differential short‐term response of functional groups to a change in forest management in a temperate forest. Forest Ecology and Management, 376, 256–264. 10.1016/j.foreco.2016.06.025

[ece370141-bib-0106] DeBano, L. F. (2000). The role of fire and soil heating on water repellency in wildland environments: A review. Journal of Hydrology, 231–232, 195–206. 10.1016/S0022-1694(00)00194-3

[ece370141-bib-0028] Dey, D. , & Schweitzer, C. (2018). A review on the dynamics of prescribed fire, tree mortality, and injury in managing oak natural communities to minimize economic loss in North America. Forests, 9, 461. 10.3390/f9080461

[ece370141-bib-0105] DRAAF Corse (Direction générale de l'alimentation, de l'agriculture et de la forêt de Corse) . (2023). Le Plan de Protection des Forêts et des Espaces naturels contre les incendies (2023–2033) . https://draaf.corse.agriculture.gouv.fr/le‐ppfeni‐2023‐2033‐a1776.html (accessed March 11, 2024).

[ece370141-bib-0030] Duane, A. , Castellnou, M. , & Brotons, L. (2021). Towards a comprehensive look at global drivers of novel extreme wildfire events. Climatic Change, 165, 43. 10.1007/s10584-021-03066-4

[ece370141-bib-0031] Eckert, M. , Gaigher, R. , Pryke, J. S. , & Samways, M. J. (2023). Short‐term arthropod recovery in eucalyptus plantations after harvesting is not affected by different residue management practices. Forest Ecology and Management, 537, 120973. 10.1016/j.foreco.2023.120973

[ece370141-bib-0032] Edlund, J. , Keramati, E. , & Servin, M. (2013). A long‐tracked bogie design for forestry machines on soft and rough terrain. Journal of Terramechanics, 50, 73–83. 10.1016/j.jterra.2013.02.001

[ece370141-bib-0033] El Khayati, M. , Chergui, B. , Barranco, P. , Fahd, S. , Ruiz, J. L. , Taheri, A. , et al. (2023). Assessing the response of different soil arthropod communities to fire: A case study from northwestern Africa. Firehouse, 6, 206. 10.3390/fire6050206

[ece370141-bib-0034] Erdmann, G. , Scheu, S. , & Maraun, M. (2012). Regional factors rather than forest type drive the community structure of soil living oribatid mites (Acari, Oribatida). Experimental & Applied Acarology, 57, 157–169. 10.1007/s10493-012-9546-9 22460402 PMC3349857

[ece370141-bib-0035] Espinosa, J. , Rodríguez de Rivera, O. , Madrigal, J. , Guijarro, M. , & Hernando, C. (2020). Predicting potential cambium damage and fire resistance in Pinus nigra Arn. Ssp. salzmannii. Forest Ecology and Management, 474, 118372. 10.1016/j.foreco.2020.118372

[ece370141-bib-0107] Fajardo‐Cantos, Á. , Peña, E. , de Las, H. J. , Plaza‐Álvarez, P. A. , González‐Romero, J. , Lucas‐Borja, M. E. , & Moya, D. (2023). Short‐term recovery of soil and pine tree canopy after late prescribed burning in a semi‐arid landscape. Science of the Total Environment, 855, 159044. 10.1016/j.scitotenv.2022.159044 36174695

[ece370141-bib-0038] Fernández, C. , Vega, J. A. , Fonturbel, T. , Jiménez, E. , & Pérez, J. R. (2008). Immediate effects of prescribed burning, chopping and clearing on runoff, infiltration and erosion in a shrubland area in Galicia (NW Spain). Land Degradation & Development, 19, 502–515. 10.1002/ldr.855

[ece370141-bib-0036] Fernandes, P. M. , & Botelho, H. S. (2003). A review of prescribed burning effectiveness in fire hazard reduction. International Journal of Wildland Fire, 12, 117. 10.1071/WF02042

[ece370141-bib-0037] Fernandes, P. M. , Davies, G. M. , Ascoli, D. , Fernández, C. , Moreira, F. , Rigolot, E. , Stoof, C. R. , Vega, J. A. , & Molina, D. (2013). Prescribed burning in southern Europe: Developing fire management in a dynamic landscape. Frontiers in Ecology and the Environment, 11, e4–e14. 10.1890/120298

[ece370141-bib-0040] Ferrat, L. , Morandini, F. , & Lapa, G. (2021). Influence of prescribed burning on a Pinus nigra subsp (Vol. 12, 915). Heat Transfer and Tree Vitality. Forests. 10.3390/f12070915

[ece370141-bib-0041] Fites‐Kaufman, J. A. , & Henson, C. J. (2004). Real time evaluation of effects of fuel treatments and other previous land management activities on fire behavior during wildfires – final report to the joint fire science program | fire research and management exchange System . https://www.frames.gov/catalog/19904

[ece370141-bib-0042] Gibson, K. S. , Johnson, N. C. , Laturno, C. , Parmenter, R. R. , & Antoninka, A. (2022). Abundance of mites, but not of collembolans or nematodes, is reduced by restoration of a Pinus ponderosa forest with thinning, mastication, and prescribed fire. Trees, Forests and People, 7, 100190. 10.1016/j.tfp.2022.100190

[ece370141-bib-0108] Gibson, K. S. , Johnson, N. C. , Laturno, C. , Parmenter, R. R. , & Antoninka, A. (2022). Abundance of mites, but not of collembolans or nematodes, is reduced by restoration of a *Pinus ponderosa* forest with thinning, mastication, and prescribed fire. Trees, Forests and People, 7, 100190. 10.1016/j.tfp.2022.100190

[ece370141-bib-0043] Gillooly, J. F. , Brown, J. H. , West, G. B. , Savage, V. M. , & Charnov, E. L. (2001). Effects of size and temperature on metabolic rate. Science, 293, 2248–2251. 10.1126/science.1061967 11567137

[ece370141-bib-0044] Gongalsky, K. B. , & Persson, T. (2013). Recovery of soil macrofauna after wildfires in boreal forests. Soil Biology and Biochemistry, 57, 182–191. 10.1016/j.soilbio.2012.07.005

[ece370141-bib-0045] Grady, K. C. , & Hart, S. C. (2006). Influences of thinning, prescribed burning, and wildfire on soil processes and properties in southwestern ponderosa pine forests: A retrospective study. Forest Ecology and Management, 234, 123–135. 10.1016/j.foreco.2006.06.031

[ece370141-bib-0046] Grodsky, S. M. , Moorman, C. E. , Fritts, S. R. , Campbell, J. W. , Sorenson, C. E. , Bertone, M. A. , Castleberry, S. B. , & Wigley, T. B. (2018). Invertebrate community response to coarse woody debris removal for bioenergy production from intensively managed forests. Ecological Applications, 28, 135–148. 10.1002/eap.1634 28949046

[ece370141-bib-0047] Gundale, M. J. , DeLuca, T. H. , Fiedler, C. E. , Ramsey, P. W. , Harrington, M. G. , & Gannon, J. E. (2005). Restoration treatments in a Montana ponderosa pine forest: Effects on soil physical, chemical and biological properties. Forest Ecology and Management, 213, 25–38. 10.1016/j.foreco.2005.03.015

[ece370141-bib-0048] Gwiazdowicz, D. J. , Kamczyc, J. , & Rakowski, R. (2011). Mesostigmatid mites in four classes of wood decay. Experimental & Applied Acarology, 55, 155–165. 10.1007/s10493-011-9458-0 21479776

[ece370141-bib-0049] Hammill, K. A. , & Bradstock, R. A. (2006). Remote sensing of fire severity in the Blue Mountains: Influence of vegetation type and inferring fire intensity | fire research and management exchange System . https://www.frames.gov/catalog/44499

[ece370141-bib-0050] Harrington, M. G. (2012). Duff mound consumption and cambium injury for centuries‐old western larch from prescribed burning in western Montana. International Journal of Wildland Fire, 22, 359–367. 10.1071/WF12038

[ece370141-bib-0051] Hättenschwiler, S. , Tiunov, A. , & Scheu, S. (2005). Biodiversity and litter decomposition in terrestrial ecosystems. Annual Review of Ecology, Evolution, and Systematics, 36, 191–218. 10.1146/annurev.ecolsys.36.112904.151932

[ece370141-bib-0052] Huebner, K. , Lindo, Z. , & Lechowicz, M. J. (2012). Post‐fire succession of collembolan communities in a northern hardwood forest. European Journal of Soil Biology, 48, 59–65. 10.1016/j.ejsobi.2011.10.004

[ece370141-bib-0053] Hunter, M. E. , & Robles, M. D. (2020). Tamm review: The effects of prescribed fire on wildfire regimes and impacts: A framework for comparison. Forest Ecology and Management, 475, 118435. 10.1016/j.foreco.2020.118435

[ece370141-bib-0111] Institut National de l'information Géographique et Forestière . (2023). Mémento – Edition 2023 . https://inventaire‐forestier.ign.fr/spip.php?article583 (accessed March 11, 2024).

[ece370141-bib-0055] Jandl, R. , Spathelf, P. , Bolte, A. , & Prescott, C. E. (2019). Forest adaptation to climate change—Is non‐management an option? Annals of Forest Science, 76, 1–13. 10.1007/s13595-019-0827-x

[ece370141-bib-0056] Jonsell, M. (2008). Saproxylic beetle species in logging residues: Which are they and which residues do they use? Norwegian Journal of Entomology, 55, 109.

[ece370141-bib-0057] Kalabokidis, K. D. , & Omi, P. N. (1998). Reduction of fire Hazard through thinning/residue disposal in the urban Interface. International Journal of Wildland Fire, 8, 29–35. 10.1071/wf9980029

[ece370141-bib-0058] Keeley, J. E. (2002). Fire Management of California Shrubland Landscapes. Environmental Management, 29, 395–408. 10.1007/s00267-001-0034-Y 11830769

[ece370141-bib-0059] Kuznetsova, N. A. (2007). Long‐term dynamics of collembolan populations in forest and meadow ecosystems. Entomological Review, 87, 11–24. 10.1134/S0013873807010022

[ece370141-bib-0060] Larrivée, M. , Drapeau, P. , & Fahrig, L. (2008). Edge effects created by wildfire and clear‐cutting on boreal forest ground‐dwelling spiders. Forest Ecology and Management, 255, 1434–1445. 10.1016/j.foreco.2007.10.062

[ece370141-bib-0061] Lazarina, M. , Devalez, J. , Neokosmidis, L. , Sgardelis, S. P. , Kallimanis, A. S. , Tscheulin, T. , Tsalkatis, P. , Kourtidou, M. , Mizerakis, V. , Nakas, G. , Palaiologou, P. , Kalabokidis, K. , Vujic, A. , & Petanidou, T. (2019). Moderate fire severity is best for the diversity of most of the pollinator guilds in Mediterranean pine forests. Ecology, 100, 1–14. 10.1002/ecy.2615 30786023

[ece370141-bib-0062] Malmström, A. (2010). The importance of measuring fire severity—Evidence from microarthropod studies. Forest Ecology and Management, 260, 62–70. 10.1016/j.foreco.2010.04.001

[ece370141-bib-0063] Malmström, A. (2012). Life‐history traits predict recovery patterns in Collembola species after fire: A 10 year study. Applied Soil Ecology, 56, 35–42. 10.1016/j.apsoil.2012.02.007

[ece370141-bib-0064] McArthur, A. G. (1967). Fire behaviour in eucalypt forests / A.G. McArthur.

[ece370141-bib-0065] McArthur, A. G. , & Cheney, N. P. (2015). The characterization of fires in relation to ecological studies. Fire Ecology, 11, 3–9. 10.1007/BF03400629

[ece370141-bib-0066] Moravec, J. , & Regeneration of N.W . (1990). African Pinus halepensis forests following fire. Vegetatio, 87, 29–36.

[ece370141-bib-0067] Moretti, M. , & Legg, C. (2009). Combining plant and animal traits to assess community functional responses to disturbance. Ecography, 32, 299–309. 10.1111/j.1600-0587.2008.05524.x

[ece370141-bib-0068] Moretti, M. , Obrist, M. , & Duelli, P. (2004). Arthropod biodiversity after forest fires: Winners and losers in the winter fire regime of the southern Alps. Ecography, 27, 173–186. 10.1111/j.0906-7590.2004.03660.x

[ece370141-bib-0069] Mott, C. M. , Hofstetter, R. W. , & Antoninka, A. J. (2021). Post‐harvest slash burning in coniferous forests in North America: A review of ecological impacts. Forest Ecology and Management, 493, 119251. 10.1016/j.foreco.2021.119251

[ece370141-bib-0070] Nadel, R. , Scholes, M. , & Byrne, M. (2007). Slash burning, faunal composition, and nutrient dynamics in a Eucalyptus grandis plantation in South Africa. Canadian Journal of Forest Research, 37, 226–235. 10.1139/X06-287

[ece370141-bib-0071] North, M. P. , Stephens, S. L. , Collins, B. M. , Agee, J. K. , Aplet, G. , Franklin, J. F. , & Fulé, P. Z. (2015). Reform forest fire management. Science, 349, 1280–1281. 10.1126/science.aab2356 26383934

[ece370141-bib-0073] Palahí, M. , Pukkala, T. , Miina, J. , & Montero, G. (2003). Individual‐tree growth and mortality models for scots pine (Pinus sylvestris L.) in north‐east Spain. Annals of Forest Science, 60, 1–10.

[ece370141-bib-0074] Paquin, P. , & Coderre, D. (1997). Deforestation and fire impact on edaphic insect larvae and other macroarthropods. Environmental Entomology, 26, 21–30. 10.1093/ee/26.1.21

[ece370141-bib-0075] Parsons, R. A. , Pimont, F. , Wells, L. , Cohn, G. , Jolly, W. M. , de Coligny, F. , Rigolot, E. , Dupuy, J. L. , Mell, W. , & Linn, R. R. (2018). Modeling thinning effects on fire behavior with STANDFIRE. Annals of Forest Science, 75, 1–10. 10.1007/s13595-017-0686-2

[ece370141-bib-0076] Pekár, S. , Michalko, R. , Loverre, P. , Líznarová, E. , & Černecká, Ľ. (2015). Biological control in winter: Novel evidence for the importance of generalist predators. Journal of Applied Ecology, 52, 270–279. 10.1111/1365-2664.12363

[ece370141-bib-0077] Perry, K. I. , Wallin, K. F. , Wenzel, J. W. , & Herms, D. A. (2018). Forest disturbance and arthropods: Small‐scale canopy gaps drive invertebrate community structure and composition. Ecosphere, 9, e02463. 10.1002/ecs2.2463

[ece370141-bib-0078] Piqué, M. , & Domènech, R. (2018). Effectiveness of mechanical thinning and prescribed burning on fire behavior in Pinus nigra forests in NE Spain. Science of the Total Environment, 618, 1539–1546. 10.1016/j.scitotenv.2017.09.316 29111258

[ece370141-bib-0079] Prichard, S. J. , Kennedy, M. C. , Wright, C. S. , Cronan, J. B. , & Ottmar, R. D. (2017). Predicting forest floor and woody fuel consumption from prescribed burns in southern and western pine ecosystems of the United States. Data in Brief, 15, 742–746. 10.1016/j.dib.2017.10.029 29124102 PMC5671462

[ece370141-bib-0080] Prunicki, M. , Kelsey, R. , Lee, J. , Zhou, X. , Smith, E. , Haddad, F. , Wu, J. , & Nadeau, K. (2019). The impact of prescribed fire versus wildfire on the immune and cardiovascular systems of children. Allergy, 74, 1989–1991. 10.1111/all.13825 31002401 PMC6801011

[ece370141-bib-0081] Raymond‐Léonard, L. J. , Bouchard, M. , & Handa, I. T. (2020). Dead wood provides habitat for springtails across a latitudinal gradient of forests in Quebec, Canada. Forest Ecology and Management, 472, 118237. 10.1016/j.foreco.2020.118237

[ece370141-bib-0082] Riechert, S. E. , & Lockley, T. (1984). Spiders as biological control agents. Annual Review of Entomology, 29, 299–320. 10.1146/annurev.en.29.010184.001503

[ece370141-bib-0083] Riera, P. , Peñuelas, J. , Farreras, V. , & Estiarte, M. (2007). Valuation of climate‐change effects on Mediterranean Shrublands. Ecological Applications, 17, 91–100. 10.1890/1051-0761(2007)017[0091:VOCEOM]2.0.CO;2 17479837

[ece370141-bib-0109] Rigolot, E. , Boivin, T. , Dreyfus, P. , Fernandez, C. , Huc, R. R. , Lefèvre, F. , Pichot, C. , & Valette, J. C. (2012). Les Pins méditerranéens Conservation, écologie, restauration et gestion : Défis dans un contexte de changements globaux. Forêt Méditerranéenne, 33, 3.

[ece370141-bib-0085] Ryan, K. C. , Knapp, E. E. , & Varner, J. M. (2013). Prescribed fire in North American forests and woodlands: History, current practice, and challenges. Frontiers in Ecology and the Environment, 11, e15–e24. 10.1890/120329

[ece370141-bib-0110] Santín, C. , & Doerr, S. H. (2016). Fire effects on soils: The human dimension. Philosophical Transactions of the Royal Society, B: Biological Sciences, 371, 20150171. 10.1098/rstb.2015.0171 PMC4874409 27216528

[ece370141-bib-0086] Santonja, M. , Fernandez, C. , Proffit, M. , Gers, C. , Gauquelin, T. , Reiter, I. M. , Cramer, W. , & Baldy, V. (2017). Plant litter mixture partly mitigates the negative effects of extended drought on soil biota and litter decomposition in a Mediterranean oak forest. Journal of Ecology, 105, 801–815. 10.1111/1365-2745.12711

[ece370141-bib-0087] Schmidt, D. A. , Taylor, A. H. , & Skinner, C. N. (2008). The influence of fuels treatment and landscape arrangement on simulated fire behavior, southern Cascade range, California. Forest Ecology and Management, 255, 3170–3184. 10.1016/j.foreco.2008.01.023

[ece370141-bib-0088] Schmidt, M. W. I. , Torn, M. S. , Abiven, S. , Dittmar, T. , Guggenberger, G. , Janssens, I. A. , Kleber, M. , Kögel‐Knabner, I. , Lehmann, J. , Manning, D. A. C. , Nannipieri, P. , Rasse, D. P. , Weiner, S. , & Trumbore, S. E. (2011). Persistence of soil organic matter as an ecosystem property. Nature, 478, 49–56. 10.1038/nature10386 21979045

[ece370141-bib-0089] Soler Martin, M. , Bonet, J. A. , Martínez De Aragón, J. , Voltas, J. , Coll, L. , & Resco De Dios, V. (2017). Crown bulk density and fuel moisture dynamics in Pinus pinaster stands are neither modified by thinning nor captured by the Forest fire weather index. Annals of Forest Science, 74, 1–11. 10.1007/s13595-017-0650-1

[ece370141-bib-0090] Stephens, S. L. , & Moghaddas, J. J. (2005). Experimental fuel treatment impacts on forest structure, potential fire behavior, and predicted tree mortality in a California mixed conifer forest. Forest Ecology and Management, 215, 21–36. 10.1016/j.foreco.2005.03.070

[ece370141-bib-0091] Tecimen, H. B. , Kavgacı, A. , & Sevgi, O. (2021). Forest fires and sustainability in the Mediterranean ecosystems. In M. Öztürk , V. Altay , & R. Efe (Eds.), Biodiversity, conservation and sustainability in Asia: Volume 1: Prospects and challenges in West Asia and Caucasus (pp. 81–100). Springer International Publishing. 10.1007/978-3-030-59928-7_5

[ece370141-bib-0092] Thévenard, L. , Leborgne, R. , & Pasquet, A. (2004). Web‐building management in an orb‐weaving spider, Zygiella x‐notata: Influence of prey and conspecifics. Comptes Rendus Biologies, 327, 84–92. 10.1016/j.crvi.2003.11.006 15015758

[ece370141-bib-0093] Thompson, H. M. , Lesser, M. R. , Myers, L. , & Mihuc, T. B. (2022). Insect community response following wildfire in an eastern North American pine barrens. Forests, 13, 66. 10.3390/f13010066

[ece370141-bib-0094] Tihay‐Felicelli, V. , Santoni, P.‐A. , Barboni, T. , & Leonelli, L. (2016). Autoignition of dead shrub twigs: Influence of diameter on ignition. Fire Technology, 52, 897–929. 10.1007/s10694-015-0514-x

[ece370141-bib-0095] Vilà‐Vilardell, L. , De Cáceres, M. , Piqué, M. , & Casals, P. (2023). Prescribed fire after thinning increased resistance of sub‐Mediterranean pine forests to drought events and wildfires. Forest Ecology and Management, 527, 120602. 10.1016/j.foreco.2022.120602

[ece370141-bib-0096] Vucic‐Pestic, O. , Birkhofer, K. , Rall, B. C. , Scheu, S. , & Brose, U. (2010). Habitat structure and prey aggregation determine the functional response in a soil predator–prey interaction. Pedobiologia, 53, 307–312. 10.1016/j.pedobi.2010.02.003

[ece370141-bib-0097] Wardhaugh, C. W. , Stone, M. J. , & Stork, N. E. (2018). Seasonal variation in a diverse beetle assemblage along two elevational gradients in the Australian wet tropics. Scientific Reports, 8, 8559. 10.1038/s41598-018-26216-8 29867113 PMC5986770

[ece370141-bib-0098] Whisenant, S. G. , Scifres, C. J. , & Ueckert, D. N. (1984). Soil water and temperature response to prescribed burning.

[ece370141-bib-0099] Wikars, L.‐O. , Sahlin, E. , & Ranius, T. (2005). A comparison of three methods to estimate species richness of saproxylic beetles (Coleoptera) in logs and high stumps of Norway spruce. The Canadian Entomologist, 137, 304–324. 10.4039/n04-104

[ece370141-bib-0100] Wikars, L.‐O. , & Schimmel, J. (2001). Immediate effects of fire‐severity on soil invertebrates in cut and uncut pine forests. Forest Ecology and Management, 141, 189–200. 10.1016/S0378-1127(00)00328-5

[ece370141-bib-0101] Zaitsev, A. S. , Gongalsky, K. B. , Persson, T. , & Bengtsson, J. (2014). Connectivity of litter islands remaining after a fire and unburnt forest determines the recovery of soil fauna. Applied Soil Ecology, 83, 101–108. 10.1016/j.apsoil.2014.01.007

